# YY2‐DRP1 Axis Regulates Mitochondrial Fission and Determines Cancer Stem Cell Asymmetric Division

**DOI:** 10.1002/advs.202207349

**Published:** 2023-06-09

**Authors:** Mankun Wei, Uli Nurjanah, Juan Li, Xinxin Luo, Rendy Hosea, Yanjun Li, Jianting Zeng, Wei Duan, Guanbin Song, Makoto Miyagishi, Vivi Kasim, Shourong Wu

**Affiliations:** ^1^ Key Laboratory of Biorheological Science and Technology Ministry of Education College of Bioengineering Chongqing University Chongqing 400044 P. R. China; ^2^ The 111 Project Laboratory of Biomechanics and Tissue Repair College of Bioengineering Chongqing University Chongqing 400044 P. R. China; ^3^ Department of Hepatobiliary and Pancreatic Oncology Chongqing University Cancer Hospital Chongqing University Chongqing 400030 P. R. China; ^4^ Life Science Innovation School of Integrative and Global Majors University of Tsukuba Tsukuba Ibaraki 305‐0006 Japan; ^5^ Chongqing Key Laboratory of Translational Research for Cancer Metastasis and Individualized Treatment Chongqing University Cancer Hospital Chongqing University Chongqing 400030 P. R. China

**Keywords:** cancer stem cells (CSCs), CSC asymmetric division, dynamin‐related protein 1 (DRP1), mitochondria fission, yin yang 2 (YY2)

## Abstract

Cancer stem cells (CSCs) are associated with tumor progression, recurrence, and therapeutic resistance. To maintain their pool while promoting tumorigenesis, CSCs divide asymmetrically, producing a CSC and a highly proliferative, more differentiated transit‐amplifying cell. Exhausting the CSC pool has been proposed as an effective antitumor strategy; however, the mechanism underlying CSC division remains poorly understood, thereby largely limiting its clinical application. Here, through cross‐omics analysis, yin yang 2 (YY2) is identified as a novel negative regulator of CSC maintenance. It is shown that YY2 is downregulated in stem‐like tumor spheres formed by hepatocarcinoma cells and in liver cancer, in which its expression is negatively correlated with disease progression and poor prognosis. Furthermore, it is revealed that YY2 overexpression suppressed liver CSC asymmetric division, leading to depletion of the CSC pool and decreased tumor‐initiating capacity. Meanwhile, YY2 knock‐out in stem‐like tumor spheres caused enrichment in mitochondrial functions. Mechanistically, it is revealed that YY2 impaired mitochondrial fission, and consequently, liver CSC asymmetric division, by suppressing the transcription of dynamin‐related protein 1. These results unravel a novel regulatory mechanism of mitochondrial dynamic‐mediated CSCs asymmetric division and highlight the role of YY2 as a tumor suppressor and a therapeutic target in antitumor treatment.

## Graphical abstract

This work revealed that yin yang 2 (YY2) triggers impaired mitochondrial fission, and subsequently promotes liver CSCs pool exhaustion, by downregulating the transcriptional activity of dynamin‐related protein 1 (DRP1). These results provide new insights regarding the regulatory mechanism of mitochondria fission‐mediated CSCs asymmetric division and highlight the potential of targeting YY2/DRP1 axis as an antitumor therapeutic strategy targeting CSCs.

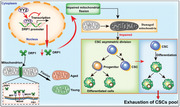

## 1 Introduction

Cancer stem cells (CSCs) are a small population of cancer cells capable of self‐renewal and tumorigenicity.^[^ ^1^ ^]^ Evidence has increasingly associated metastasis, chemoresistance, and recurrence of cancer with the presence of CSCs.^[^ ^2^, ^3^, ^4^ ^]^ To maintain a stable pool while also promoting tumorigenesis and generating a heterogeneous population of more differentiated cells, CSCs divide asymmetrically, thereby producing a daughter cell that maintains self‐renewal along with other CSC properties, and a highly proliferative, more differentiated transit‐amplifying cell.^[^ ^5^, ^6^ ^]^ Such asymmetric division allows CSCs to generate numerous more differentiated cells during the lifetime of an individual, promoting tumor growth and progression.^[^ ^7^ ^]^ Disrupting asymmetric division can decrease the number of CSCs by altering the balance between self‐renewal and differentiation in CSCs, thereby decreasing tumor initiation and tumor growth potentials, while increasing drug sensitivity.^[^ ^8^, ^9^ ^]^ Hence, exhausting the CSC pool by targeting asymmetric division and inducing CSC differentiation has been proposed as a potential antitumor strategy.^[^ ^10^, ^11^, ^12^, ^13^ ^]^

Recent studies have shed light on the regulatory processes underlying the asymmetric division of CSCs, including progression through the cell cycle, which has been linked to impaired self‐renewal, tumorigenesis, and drug resistance.^[^ ^14^ ^]^ Another mechanism involves mitochondrial fission and asymmetric segregation, whereby young, healthy mitochondria are generated and distributed asymmetrically to the two daughter cells. Consequently, the cell that receives fewer relatively dysfunctional mitochondria (hereafter referred to as “dysfunctional mitochondria”) maintains its stemness; whereas the cell that receives fewer relatively “healthy” mitochondria (hereafter referred to as “healthy” mitochondria) loses its stemness.^[^ ^15^, ^16^, ^17^ ^]^ However, the regulatory mechanism of CSC asymmetric division remains poorly understood, limiting therapeutic applications.

Transcription factors play a critical role in regulating various pathways, including development and differentiation.^[^ ^18^ ^]^ Aberrant expression of many transcription factors that direct developmental decisions can increase their oncogenicity by promoting tumor initiation, progression, and metastasis.^[^ ^19^, ^20^, ^21^ ^]^ Not surprisingly, transcription factor homeostasis is crucial for CSC maintenance. Disruption of this balance can result in changes in the expression of various stemness‐related genes.^[^ ^20^ ^]^ Despite their importance in maintaining the CSC pool, the role of transcription factors in asymmetric division of CSCs remains unclear.

This study aimed to elucidate the regulatory mechanism responsible for maintaining stemness in CSCs. Using RNA sequencing (RNA‐Seq), we identified the zinc‐finger transcription factor yin yang 2 (YY2) as being poorly expressed in liver CSCs. Systematic investigations elucidated that YY2 suppressed liver CSC asymmetric division, subsequently decreasing tumor‐initiating capability. Furthermore, our study revealed that YY2 suppressed dynamin‐related protein 1 (DRP1)‐mediated mitochondrial fission, which led to depletion of the liver CSC pool and subsequent decrease in tumor‐initiating capability. Together, our findings disclose a novel regulatory mechanism of mitochondrial fission‐mediated CSC asymmetric division, thereby providing new perspective regarding CSC maintenance as well as a new paradigm for antitumor treatment.

## 2 Results

### 2.1 YY2 is Downregulated in Liver CSCs

**Figure 1:**
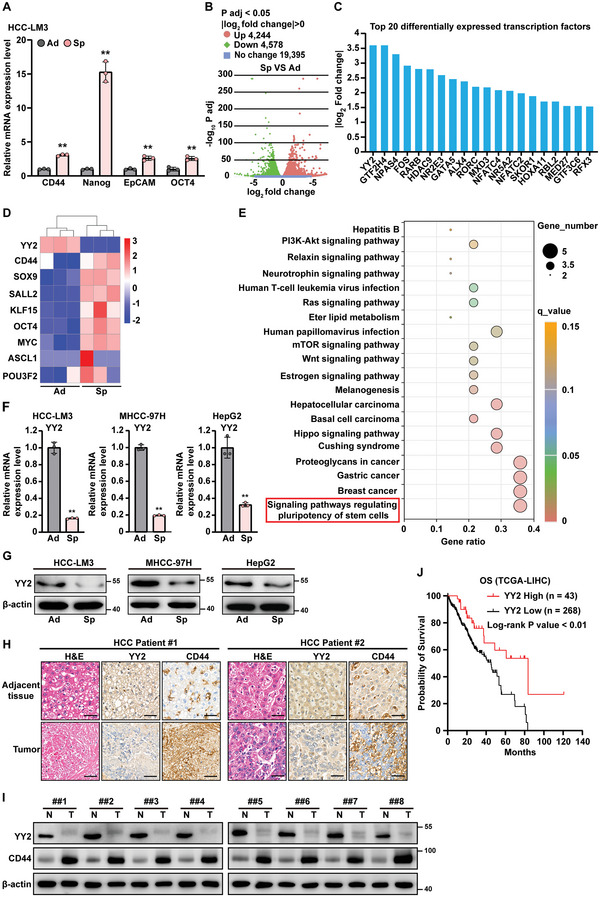
YY2 negatively correlated with CSC and HCC progression. A) mRNA expression levels of CD44, Nanog, EpCAM, and OCT4 in adherent and stem‐like tumor spheres formed by HCC‐LM3 cells, as determined using qRT‐PCR. B) Volcano plot of log_2_ fold‐change versus adjusted *p‐*value for gene expression changes in adherent and tumor spheres formed by HCC‐LM3 cells, as analyzed by RNA‐seq. C) Fold‐change of top 20 differentially expressed transcription factors in adherent and stem‐like tumor spheres formed by HCC‐LM3 cells. D) Heatmap showing the expression of YY2 and differently expressed stemness‐related genes significantly enriched in tumor spheres formed by HCC‐LM3 cells. E) KEGG analysis of differentially expressed genes in YY2‐overexpressed cells using GSE184138 database. The top 20 pathways enriched in YY2‐overexpressed cells are shown. F,G) YY2 mRNA (F) and protein (G) expression levels in adherent and stem‐like tumor spheres formed by HCC‐LM3, MHCC‐97H, and HepG2 cells, as determined using qRT‐PCR and western blotting, respectively. H,I) YY2 and CD44 expression level in clinical HCC tissues and the corresponding normal adjacent tissue, as analyzed using immunohistochemical staining (H) and western blotting (I); Scale bars: 50 µm. J) Kaplan–Meier plot of overall survival (OS) in clinical HCC patients with low and high YY2 expression as obtained from the TCGA dataset (*n* = 311; *p* < 0.01). *β*‐actin was used for qRT‐PCR normalization and as western blotting loading control. Ad: adherent cells; Sp: stem‐like tumor spheres; LIHC: liver hepatocellular carcinoma. Quantification data are shown as mean ± SD (*n* = 3); *p* values were calculated using two‐tailed unpaired Student's *t*‐test. For experiments using clinical samples, *p* values were calculated using one‐way ANOVA. ^**^ *p* < 0.01.

To identify novel regulators responsible for CSC maintenance, we first generated stem‐like tumor spheres using three hepatocellular carcinoma (HCC) cell lines: HCC‐LM3, MHCC‐97H, and HepG2. Enrichment with CSCs in the tumor spheres was confirmed by an increase in the CSC markers CD44, Nanog, EpCAM, and OCT4 (**Figure** **1**A; Figure S1A,B, Supporting Information). Differentially expressed genes were then identified by RNA‐Seq using adherent and stem‐like tumor spheres formed by HCC‐LM3 cells. Among the 28217 detected genes, 8822 were differentially expressed in stem‐like tumor spheres. Of these, 4244 genes were upregulated and 4578 genes were downregulated (Figure 1B).

To reveal potential transcription factors involved in CSC maintenance, we extracted the top 20 transcription factors differentially expressed in stem‐like tumor spheres and identified two transcriptional factors, YY2 and GTF2H4, as having the most altered expression (Figure 1C). We next validated the expression levels of these two factors, and the results showed a higher fold‐change of YY2 expression in adherent and stem‐like tumor spheres compared to GTF2H4 (Figure S1C, Supporting Information). Given that YY2 has been reported to regulate mouse embryonic stem cell commitment into cardiovascular lineage,^[^ ^22^ ^]^ we chose to explore its relation with CSC maintenance in this study. Heatmap analysis of gene expression in adherent cells and stem‐like tumor spheres (Figure 1D) revealed a negative correlation between YY2 and stem‐related factors, including CD44, SOX9, SALL2, KLF15, OCT4 (also known as POU class 5 homeobox 1 or POU5F1), MYC, ASCL1, and POU3F2. To further confirm this correlation, we performed an enrichment analysis for differentially expressed genes in YY2‐overexpressed cells based on the Kyoto Encyclopedia of Genes and Genomes (KEGG) using RNA‐Seq data obtained from our previous study (https://www.ncbi.nlm.nih.gov; GSE184138).^[^ ^23^ ^]^ Accordingly, “signaling pathways regulating pluripotency” was significantly enriched upon YY2 overexpression (Figure 1E). Downregulation of YY2 mRNA and protein levels in stem‐like tumor spheres compared to adherent cells was further validated in HCC cell lines (Figure 1F,G). Together, these results pointed to enrichment of YY2‐downregulated cells in stem‐like tumor spheres, suggesting a negative correlation between YY2 and CSCs.

CSCs are essential for tumor initiation and progression, thereby leading to high mortality and recurrence rates, as well as low therapeutic sensitivity and overall survival.^[^ ^6^ ^]^ Using clinical samples obtained from patients with HCC, we confirmed the downregulation of YY2 in tumor tissues compared to corresponding adjacent tissues, along with a negative correlation between YY2 and CD44 (Figure 1H,I). The Cancer Genome Atlas (TCGA) database revealed that YY2 was downregulated in HCC patients according to disease progression (TCGA dataset for liver HCC, *n* = 304; Figure S1D, Supporting Information). Furthermore, overall survival was significantly higher in HCC patients with high YY2 expression than in those with low YY2 expression (TCGA, *n* = 311; Figure 1J). Together, these results suggest a negative correlation between YY2 and CSCs, as well as between YY2 and HCC progression.

### 2.2 YY2 Modulates Tumor Initiation through Regulation of Liver CSCs

**Figure 2:**
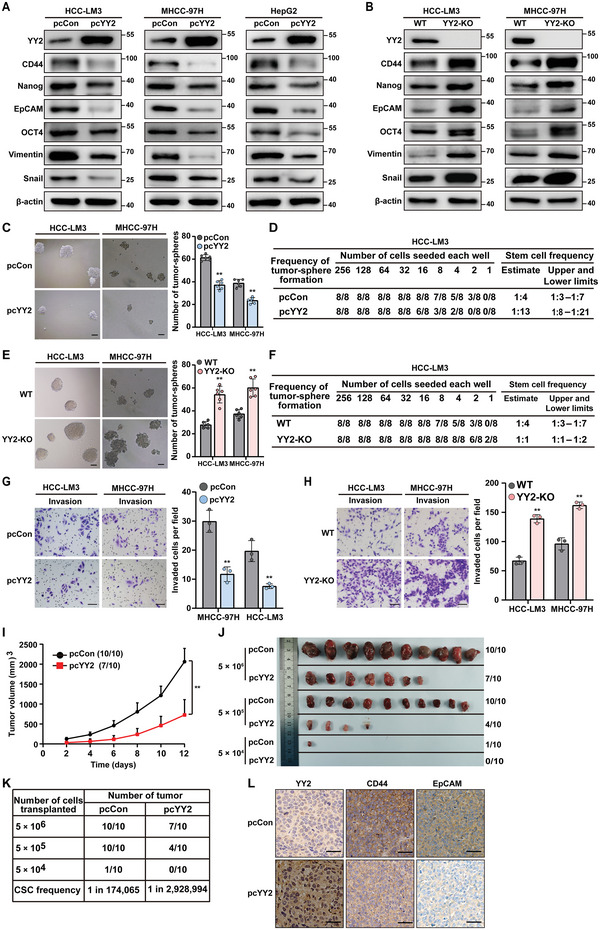
YY2 modulates tumor‐initiating capacities by regulating CSCs. A,B) Protein expression levels of CSC markers in YY2‐overexpressed (A) and YY2 knock‐out (B) HCC cells, as determined by western blotting. C,D) Tumor sphere formation potential (C) and CSC frequency (D) in YY2‐overexpressed HCC cells, as determined using in vitro LDA. Representative images (left; scale bars: 200 µm) and quantification results (right; *n* = 6) are shown. E,F) Tumor sphere formation potential (E) and CSC frequency in YY2 knock‐out HCC cells (F), as determined using in vitro LDA. Representative images (left; scale bars: 200 µm) and quantification results (right; *n* = 6) are shown. G,H) Invasion capacity of YY2‐overexpressed (G) and YY2 knock‐out (H) HCC cells. Representative images (scale bars: 100 µm) and quantification results from three independent experiments (*n* = 6 per experiment) are shown. I–K) Tumor‐initiating potential of YY2‐overexpressed HCC‐LM3 cells, as examined by in vivo LDA using xenograft experiment. Tumor volume (I), morphological images (J), and CSC frequencies (K) are shown. The ratio of the number of mice with tumor to the number of total mice transplanted with indicated cells is shown. L) Immunohistochemical staining images against YY2, CD44, and EpCAM in the tissue sections of xenografted tumors formed by YY2‐overexpressed HCC‐LM3 cells (scale bars: 50 µm). Cells transfected with pcCon or corresponding wild‐type cells were used as controls. *β*‐actin was used as western blotting loading control. Quantification data are shown as mean ± SD. *p* values were calculated using two‐tailed unpaired Student's *t*‐test. For xenograft experiments, *p* values were calculated using one‐way ANOVA. pcCon: pcEF9‐Puro; ^**^ *p* < 0.01.

To determine the role of YY2 in liver CSCs, we first confirmed the expression of a previously constructed YY2 overexpression vector^[^ ^23^ ^]^ in HCC‐LM3 cells (Figure S2A,B, Supporting Information), and then examined its effect on CSC markers. YY2 overexpression decreased the mRNA levels of CD44, Nanog, EpCAM, and OCT4, as well as epithelial‐mesenchymal transition markers Vimentin and Snail, in HepG2 cells (Figure S2C, Supporting Information). Furthermore, it reduced protein expression of these CSC markers in each cell line (**Figure** **2**A). Meanwhile, knocking out YY2 in HCC‐LM3 (HCC‐LM3^YY2KO^) and MHCC‐97H (MHCC‐97H^YY2KO^) cells using CRISPR/Cas9 technology (Figure S2D,E, Supporting Information) resulted in robust upregulation of CSC markers (Figure 2B).

Next, we examined the role of YY2 in regulating tumor sphere formation and tumor initiation. YY2 overexpression clearly decreased the number of tumor spheres formed by HCC‐LM3 and MHCC‐97H and cells (Figure 2C), as well as the frequency of liver CSCs in the spheres from 1:4 to 1:13 (Figure 2D) and 1:11 (Figure S3A, Supporting Information), respectively. In contrast, knocking out YY2 robustly increased the number of tumor spheres formed by HCC‐LM3 and MHCC‐97H cells (Figure 2E), as well as the frequency of liver CSCs from 1:4 to 1:1 (Figure 2F; Figure S3B, Supporting Information). Furthermore, YY2 overexpression decreased the size of anchorage‐dependent colonies formed by HCC cells (Figure S3C, Supporting Information).

Altered YY2 expression also correlated negatively with other hallmarks of CSCs. YY2 overexpression markedly reduced the migration and invasion potential of HCC cells (Figure 2G; Figure S4A, Supporting Information); whereas YY2 silencing had the opposite effect (Figure 2H; Figure S4B–D, Supporting Information). Additionally, YY2 overexpression decreased the resistance of HCC cells to cisplatin, an antitumor drug commonly used for treating patients with HCC, as indicated by a lower half‐inhibitory concentration (IC_50_; Figure S4E, Supporting Information).

Subsequently, we examined the tumor‐initiating capacity using a xenograft assay with HCC‐LM3 cells stably overexpressing YY2 (Figure S5A, Supporting Information). Morphological analysis revealed that YY2 overexpression clearly suppressed tumor growth (Figure 2I; Figure S5B, Supporting Information). Meanwhile, in vivo limiting dilution assay (LDA) performed by xenograft experiment using a series of cell amounts as indicated revealed that YY2 overexpression decreased liver CSC frequency by > 10‐fold (Figure 2J,K), as well as the expression of CSC markers CD44 and EpCAM in the xenografted tumor lesions (Figure 2L; Figure S5C, Supporting Information). Together, these results demonstrate for the first time that YY2 is a novel regulator of CSC stemness which could suppress the frequency of CSCs, thereby attenuating their tumor‐initiating capacity.

### 2.3 YY2 Suppresses Liver CSC Asymmetric Division

**Figure 3:**
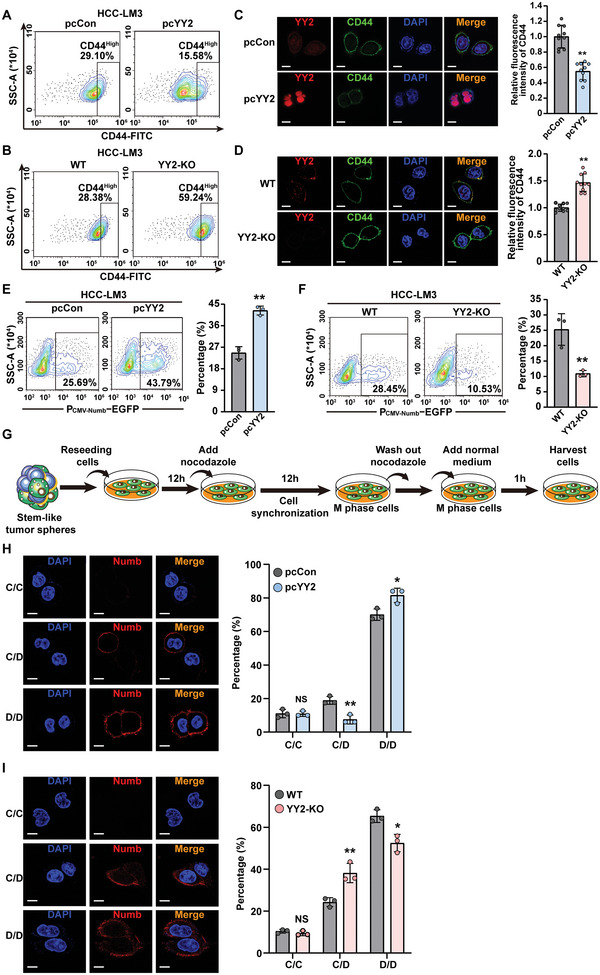
YY2 suppresses CSC asymmetric division. A,B) Percentage of CD44^High^ cells in YY2‐overexpressed (A) and YY2 knock‐out (B) HCC‐LM3 cells, as evaluated using flow cytometry. C,D) CD44 fluorescence intensity in YY2‐overexpressed (C) and YY2 knock‐out (D) HCC‐LM3 cells. Representative images (scale bars: 10 µm) and quantification results (*n* = 10) are shown. E,F) Percentages of EGFP‐positive cells in YY2‐overexpressed (E) and YY2 knock‐out (F) HCC‐LM3 cells transfected with P_CMV‐Numb_‐EGFP, as analyzed using flow cytometry (*n* = 3). G) Schematic diagram of cell‐cycle synchronization in M phase using nocodazole (final concentration: 100 ng mL^−1^). H,I) Sphere cell division types in YY2‐overexpressed (H) and YY2 knock‐out (I) HCC‐LM3 cells. Immunofluorescence of Numb (red) and DAPI (blue) representing three division types: CSC/CSC (C/C; Numb^−^/Numb^−^), CSC/non‐CSC (C/D; Numb^−^/Numb^+^), and non‐CSC/non‐CSC (D/D; Numb^+^/Numb^+^). Representative images (left; scale bars: 10 µm) and quantification results from three independent experiments (right, each dot represents 24 to 32‐pairs of daughter cells) are shown. Cells transfected with pcCon or wild‐type cells were used as controls. Quantification data are shown as mean ± SD. *p* values were calculated using two‐tailed unpaired Student's *t*‐test. pcCon: pcEF9‐Puro; ^*^ *p* < 0.05; ^**^ *p* < 0.01; ns: not significant.

Asymmetric division maintains the pool of CSCs while also generating highly proliferative non‐CSC cells.^[^ ^5^, ^6^ ^]^ YY2 overexpression markedly decreased the percentage of CD44^High^ HCC‐LM3 cells (**Figure** **3**A); whereas YY2 knock‐out caused a conspicuous increase (Figure 3B). These results were further confirmed by immunofluorescence staining, whereby CD44 fluorescence intensity correlated negatively with YY2 expression (Figure 3C,D).

We next tried to trace the fate of liver CSCs by establishing stable HCC‐LM3 cells overexpressing EGFP under the promoters of CMV‐Numb and CMV‐Albumin (Figure S6A,B, Supporting Information), as described previously.^[^ ^24^, ^25^, ^26^, ^27^ ^]^ As reported previously, Numb could mark the non‐CSC daughter cells, while Albumin expression increased in more differentiated hepatocytes; and thus, they could be used as a marker of CSC asymmetric division.^[^ ^7^, ^14^, ^25^, ^26^, ^28^, ^29^ ^]^ We observed that compared to those enriched in stem‐like tumor spheres, adherent HCC‐LM3 cells showed significantly higher percentages of GFP‐positive cells driven by CMV‐Numb (Figure S6C, Supporting Information) and CMV‐Albumin (Figure S6D, Supporting Information). In contrast to CD44, YY2 overexpression increased the percentage of EGFP‐positive HCC‐LM3 cells transfected with P_CMV‐Numb_‐EGFP or P_CMV__‐__Alb_‐EGFP (Figure 3E; Figure S6E, Supporting Information); whereas YY2 knock‐out decreased them (Figure 3F; Figure S6F, Supporting Information). Furthermore, YY2 overexpression increased Numb and albumin protein expression levels, while YY2 knock‐out decreased them (Figure S6G,H, Supporting Information). Taken together, these results demonstrated that YY2 overexpression enhanced CSCs differentiation and thereby increasing the population of non‐CSCs.

We next assessed whether YY2 affected CSC asymmetric division by analyzing Numb expression in two progenies originating from a single parental cell. To this end, cells obtained from stem‐like tumor spheres were synchronized in the mitotic phase using nocodazole. Nocodazole was then washed to allow cell division and 1 h later, cells were harvested and stained with an anti‐Numb antibody (Figure 3G).^[^ ^14^ ^]^ Asymmetric division gives rise to a Numb‐negative cell that maintains stemness and a Numb‐positive, non‐CSC daughter cell. Instead, symmetric division gives rise to two Numb‐negative CSCs or two Numb‐positive non‐CSCs. As shown in Figure 3H, YY2 overexpression clearly suppressed asymmetric division and favored the generation of two non‐CSC daughter cells; whereas the opposite was observed in HCC‐LM3^YY2KO^ cells (Figure 3I). Notably, neither YY2 overexpression nor YY2 knock‐out altered the symmetric division into two liver CSC daughter cells. This finding suggested that, while YY2 suppressed liver CSC asymmetric division in favor of a symmetric one that generated two more differentiated cells, it did not affect the symmetric production of two CSCs. Taken together, these results point to YY2 as a novel regulator of CSC asymmetric division and thereby, CSC fate.

### 2.4 YY2 Suppresses CSC Stemness by Inhibiting Mitochondrial Fission

**Figure 4:**
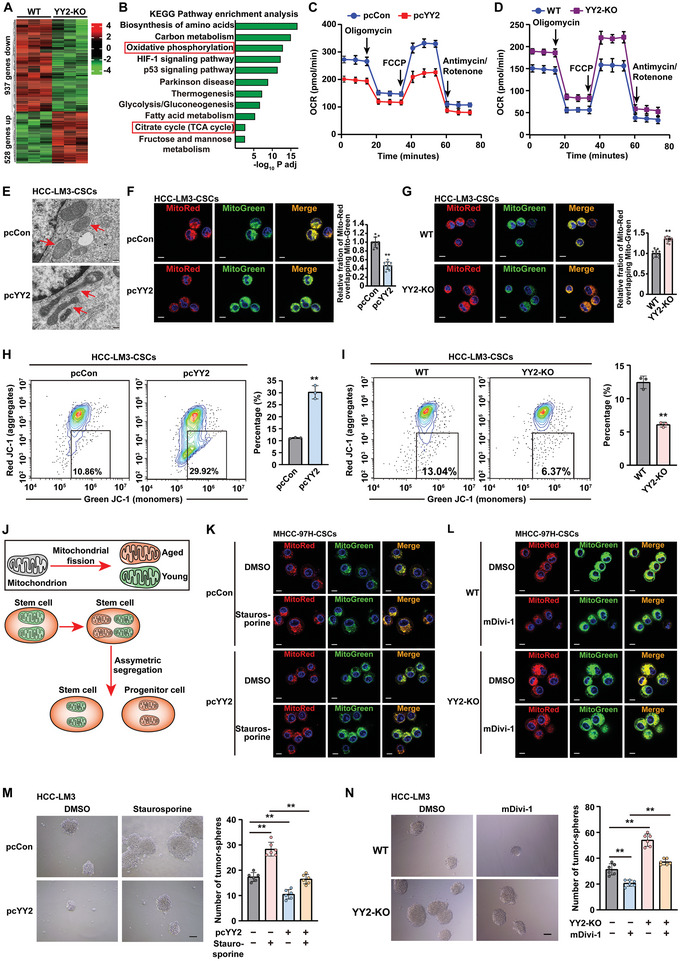
YY2 suppresses CSC stemness by inhibiting mitochondrial fission. A) Heatmap showing differentially expressed genes in MHCC‐97H^YY2KO^ stem‐like tumor spheres. B) KEGG analysis of differentially expressed genes in MHCC‐97H^YY2KO^ stem‐like tumor spheres with adjusted *p‐*value < 0.05. C,D) OCR of YY2‐overexpressed (C) and YY2 knock‐out (D) HCC‐LM3 stem‐like tumor spheres (*n* = 3). E) Transmission electron microscopy images of mitochondria in YY2‐overexpressed HCC‐LM3 stem‐like tumor spheres. Scale bars: 200 nm. F,G) Δ*Ψ*_m_ in YY2‐overexpressed (F) and YY2 knock‐out (G) HCC‐LM3 stem‐like tumor spheres, as examined using MitoTracker Red/MitoTracker Green staining. Representative images (left; scale bars: 10 µm) and quantification results (right; *n* = 10) are shown. H,I) Δ*Ψ*_m_ in YY2‐overexpressed (H) and YY2‐knock‐out (I) HCC‐LM3 stem‐like tumor spheres, as determined using JC‐1 staining and flow cytometry (*n* = 3). J) Schematic diagram of mitochondrial fission‐regulated stem cells asymmetric division. (K and L) Δ*Ψ*_m_ in YY2‐overexpressed HCC‐LM3 stem‐like tumor spheres treated with staurosporine K), and HCC‐LM3^YY2KO^ stem‐like tumor spheres treated with mDivi‐1 L), as examined using MitoTracker Red/MitoTracker Green staining (scale bars: 10 µm). M,N) Tumor sphere formation potential of YY2‐overexpressed HCC‐LM3 cells treated with staurosporine (M), and HCC‐LM3^YY2KO^ cells treated with mDivi‐1 (N). Representative images (left; scale bars: 200 µm) and quantification results (right; *n* = 6) are shown. Cells transfected with pcCon or corresponding wild‐type cells were used as controls. The final concentrations of staurosporine and mDivi‐1 used were 1 *µ*M and 10 *µ*M, respectively. Quantification data are shown as mean ± SD. *p* values were calculated using two‐tailed unpaired Student's *t*‐test. pcCon: pcEF9‐Puro; FCCP: carbonyl cyanide‐p‐trifluoromethoxyphenylhydrazone; ^**^ *p* < 0.01.

To determine the molecular mechanism responsible for maintaining CSC stemness, we performed RNA‐Seq analysis using stem‐like tumor spheres formed by MHCC‐97H and MHCC‐97H^YY2KO^ cells and identified YY2‐regulated genes. Compared to wild‐type stem‐like tumor spheres, 1465 genes were differentially expressed in YY2 knock‐out tumor spheres: 528 of them were upregulated and 937 were downregulated (**Figure** **4**A). KEGG analysis showed that “oxidative phosphorylation” and “citrate cycle (TCA cycle)” were enriched in YY2 knock‐out stem‐like tumor spheres (Figure 4B), hinting at a possible regulatory role of YY2 in mitochondrial function. This possibility was confirmed by a low oxygen consumption rate (OCR) in stem‐like tumor spheres with elevated YY2 levels (Figure 4C), and a high OCR in stem‐like tumor spheres formed by HCC‐LM3^YY2KO^ cells (Figure 4D). Furthermore, transmission electron microscopy revealed increases of dysfunctional, “spaghetti‐like” appearance of mitochondria in stem‐like tumor spheres formed by HCC‐LM3 cells overexpressing YY2 (Figure 4E; Figure S7A, Supporting Information), and more fragmented, less tubular mitochondria in those formed by HCC‐LM3^YY2KO^ cells (Figure S7B, Supporting Information), indicating that YY2 increased dysfunctional mitochondria. As membrane potential reflects the condition of mitochondria, we next examined the effect of YY2 on mitochondrial membrane potential (Δ*Ψ*_m_). Staining with MitoTracker Red, a Δ*Ψ*_m_‐dependent mitochondria marker, and MitoTracker Green, a Δ*Ψ*_m_‐independent mitochondria marker, revealed that YY2 overexpression clearly reduced Δ*Ψ*_m_ in stem‐like tumor spheres formed by HCC‐LM3 and MHCC‐97H cells (Figure 4F; Figure S7C, Supporting Information). In contrast, YY2 knock‐out significantly increased these levels (Figure 4G; Figure S7D, Supporting Information). A similar result was obtained with JC‐1, a mitochondrial marker that presents as green fluorescent monomers in the cytoplasm and as red fluorescent aggregates in healthy mitochondria with high Δ*Ψ*_m_. YY2 overexpression increased the proportion of HCC‐LM3 and MHCC‐97H stem‐like tumor spheres containing elevated green fluorescence or cells with dysfunctional mitochondria (Figure 4H; Figure S7E, Supporting Information); whereas YY2 knock‐out decreased them (Figure 4I; Figure S7F, Supporting Information). These results revealed that YY2 enhanced the number of dysfunctional mitochondria, while lowering that of healthy mitochondria, leading to impaired mitochondrial function.

Mitochondrial fission produces a dysfunctional and healthy mitochondrion.^[^ ^30^ ^]^ During asymmetric division, stem cell progeny receiving fewer healthy mitochondria lost its stemness, while the one receiving fewer dysfunctional mitochondria maintained it (Figure 4J). Hence, we examined whether YY2 augmented the ratio of cells with dysfunctional mitochondria and suppressed tumor stemness by regulating mitochondrial fission. Treatment with staurosporine, a mitochondrial fission inducer,^[^ ^31^ ^]^ restored the Δ*Ψ*_m_ in stem‐like tumor spheres formed by YY2‐overexpressed MHCC‐97 cells (Figure 4K; Figure S8A–C, Supporting Information). In contrast, treatment with mDivi‐1, a dynamin inhibitor that blocks mitochondrial fission, suppressed the increase in Δ*Ψ*_m_ observed in stem‐like tumor spheres formed by MHCC‐97H^YY2KO^ cells (Figure 4L; Figure S8D–F, Supporting Information), suggesting that YY2 prevented mitochondrial fission. Furthermore, staurosporine treatment conspicuously increased the formation of stem‐like tumor spheres, and restored the number of stem‐like tumor spheres in YY2‐overexpressed HCC‐LM3 and MHCC‐97H cells (Figure 4M; Figure S9A,B, Supporting Information). Instead, treatment with mDivi‐1 inhibited the increase in stem‐like tumor spheres seen in YY2 knock‐out HCC‐LM3 cells (Figure 4N), and similarly, in YY2 knock‐out MHCC‐97H cells (Figure S9C,D, Supporting Information). Together, these results indicate that YY2 negatively regulates tumor stemness by suppressing mitochondrial fission.

### 2.5 YY2 is a Transcriptional Suppressor of DRP1

**Figure 5:**
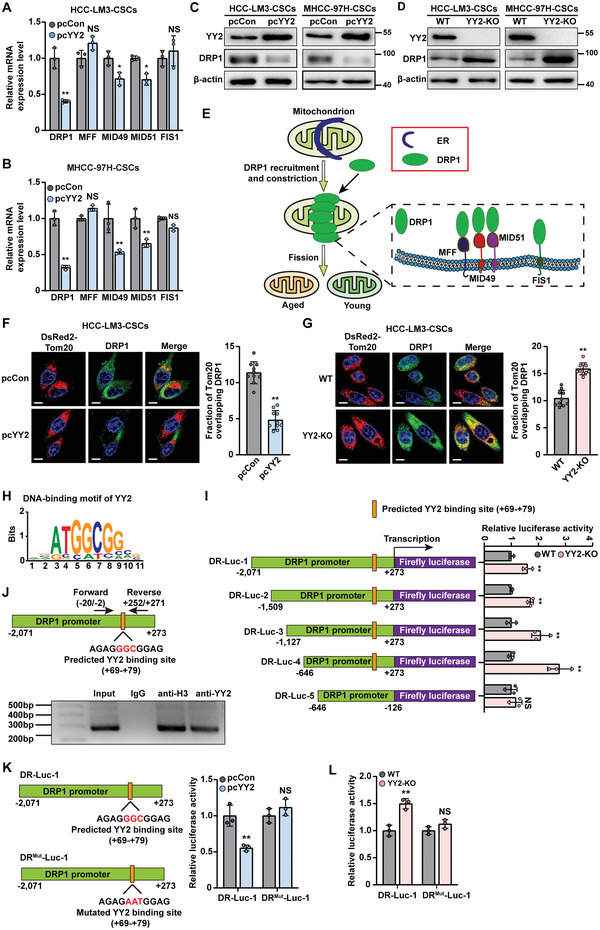
YY2 directly binds to DRP1 promoter and regulates its transcriptional activity. A,B) mRNA expression levels of mitochondrial fission‐related genes in stem‐like tumor spheres formed by YY2‐overexpressed HCC‐LM3 (A) and MHCC‐97H (B) cells, as determined using qRT‐PCR. C,D) DRP1 protein expression levels in stem‐like tumor spheres formed by YY2‐overexpressed (C) and YY2 knock‐out (D) HCC cells, as examined using western blotting. E) Schematic diagram of mitochondrial fission. F,G) Distribution of DRP1 on mitochondria in YY2‐overexpressed (F) and YY2 knock‐out (G) HCC‐LM3 stem‐like tumor spheres, as examined using mitochondrial membrane marker DsRed2‐Tom20. Representative images (left; scale bars: 10 µm) and quantification results (right; *n* = 10) are shown. H) DNA‐binding motif of YY2 on DRP1 promoter, as predicted using JASPAR. I) Relative activities of DRP1 promoter reporter vectors (DR‐Lucs) in HCC‐LM3^YY2KO^ cells, as analyzed using dual luciferase reporter assay. J) Binding capacity of YY2 to the predicted region in the DRP1 promoter region, as determined using ChIP assay followed by PCR. The location of the primer pair used for PCR is shown. K,L) Relative activities of DR‐Luc‐1 reporter vector with mutated YY2 predicted binding site (DRP1‐Luc‐1^Mut^) in YY2‐overexpressed (K) and YY2 knock‐out (L) HCC‐LM3 cells, as analyzed using dual luciferase reporter assay. Mutated nucleotides are shown in red. *β*‐actin was used for qRT‐PCR normalization and as western blotting loading control. Cells transfected with pcCon or corresponding wild‐type cells were used as controls. Quantification data are shown as mean ± SD (n = 3, unless otherwise indicated). *p* values were calculated using two‐tailed unpaired Student's *t*‐test. pcCon: pcEF9‐Puro; ^*^ *p* < 0.05; ^**^ *p* < 0.01; ns: not significant.

Next, we explored the molecular mechanism underlying the regulation of mitochondrial fission by YY2. To this end, we analyzed the levels of mitochondrial fission‐related genes in stem‐like tumor spheres formed by HCC‐LM3 and MHCC‐97H cells with altered YY2 expression. YY2 overexpression robustly decreased DRP1 mRNA levels (**Figure** **5**A,B); whereas YY2 knock‐out caused a significant increase (Figure S10A,B, Supporting Information). The negative regulation of DRP1 by YY2 in stem‐like tumor spheres was confirmed by DRP1 protein expression (Figure 5C,D).

DRP1 is usually localized in the cytosol and must be recruited to the surface of mitochondria to mediate constriction and scission (Figure 5E). To assess the subcellular localization of DRP1, we overexpressed DsRed2‐marked Tom20, a mitochondrial membrane marker. DRP1 colocalization with Tom20 was low in YY2‐overexpressed HCC‐LM3 cells (Figure 5F) and high in YY2 knock‐out cells (Figure 5G) obtained from stem‐like tumor spheres, indicating that YY2 suppressed DRP1 distribution in the mitochondrial network of liver CSCs. Together, these results suggested that YY2 negatively regulated DRP1 expression, which in turn affected its activity and distribution.

Using JASPAR (https://www.jaspar.genereg.net), we predicted YY2 consensus binding site at +69 to +79 of the DRP1 promoter (Figure 5H), and constructed a series of luciferase reporters coupled to different fragments of the DRP1 promoter (Figure 5I). YY2 knock‐out significantly increased the activities of DR‐Luc‐1, DR‐Luc‐2, DR‐Luc‐3, and DR‐Luc‐4, which contained the −2071 to +273, −1509 to +273, −1127 to +273, and −646 to +273 regions of the DRP1 promoter, respectively (Figure 5I); whereas YY2 overexpression suppressed them (Figure S10C, Supporting Information). These findings confirmed the potential role of YY2 in regulating DRP1 transcription. Importantly, neither YY2 overexpression nor knock‐out had any significant effect on the activity of DR‐Luc‐5, which did not harbor the predicted YY2 binding site, suggesting that the −125 to +273 region of DRP1 promoter was crucial for its transcriptional regulation by YY2.

To further assess whether YY2 could regulate DRP1 transcription directly, we then analyzed whether YY2 bound to the predicted site within the DRP1 promoter by performing a chromatin immunoprecipitation (ChIP) assay with primer pair flanking the +69 to +79 region. The corresponding promoter region was detected by an anti‐YY2 antibody (Figure 5J), indicating that YY2 could bind directly to the −20 to +271 region of the DRP1 promoter. Finally a luciferase reporter assay using DR^Mut^‐Luc‐1, a DR‐Luc‐1 reporter with three mutated nucleotides in the predicted YY2‐binding site (GGC to AAT; Figure 5K), revealed that mutations in the YY2 binding site diminished both the suppressive effect of YY2 overexpression and the stimulatory effect of YY2 knock‐out on wild‐type DR‐Luc‐1 (Figure 5K,L). Similarly, mutation of the 232^nd^ amino acid in YY2 from proline to leucine abolished the YY2‐dependent suppressive effect on DR‐Luc‐1 (Figure S10D,E, Supporting Information); whereas mutations of the 260^th^ glycine to alanine and the 212^nd^ glutamic acid to lysine had no significant effect. Accordingly, the transcriptional repressor region of YY2 appeared crucial for its regulation of DRP1 transcription. This effect was also confirmed at mRNA and protein levels, as overexpressing YY2^P232L^ mutant failed to suppress DRP1 mRNA and protein levels (Figure S10F,G, Supporting Information). Together, these results indicate that YY2 can directly suppress DRP1 transcription by binding to the DRP1 promoter, most plausibly at its consensus sequence in the +69 to +79 region.

### 2.6 DRP1 is Crucial for YY2 Regulation of Mitochondrial Fission

**Figure 6:**
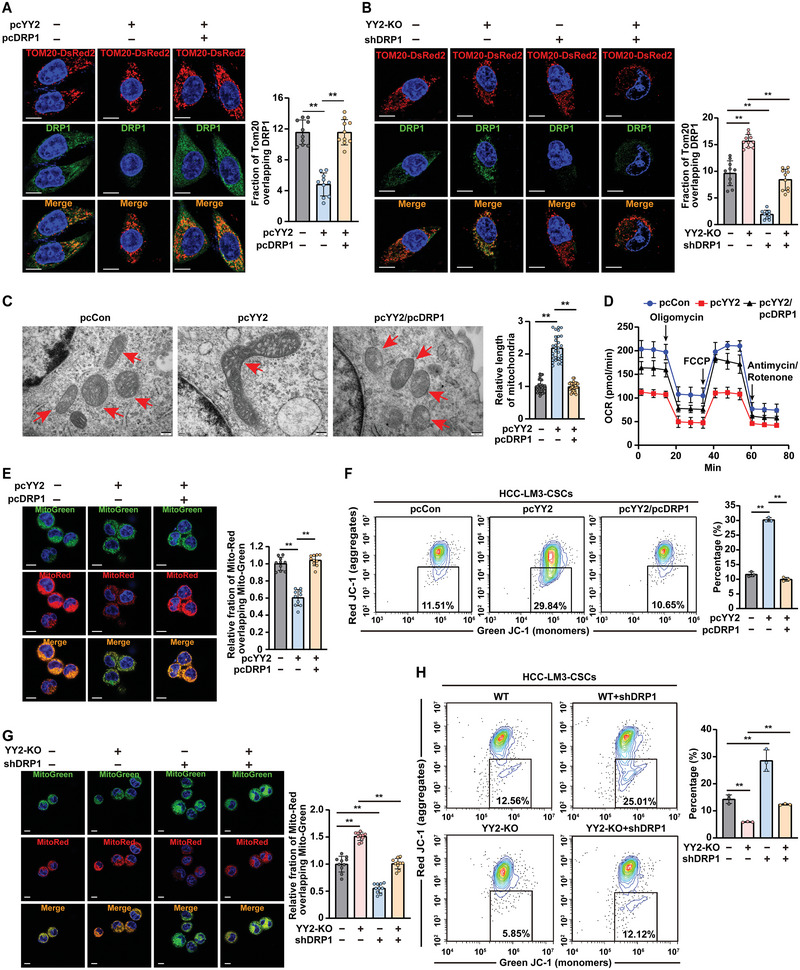
DRP1 is crucial for YY2 regulation on mitochondrial fission. A,B) Distribution of DRP1 in mitochondria in YY2‐overexpressed, DRP1‐overexpressed HCC‐LM3 cells (A) and DRP1 knock‐down HCC‐LM3^YY2KO^ cells (B) was analyzed using DsRed‐Tom20. Representative images (scale bars: 10 µm) and quantification results (right; *n* = 10) are shown. C) Transmission electron microscopy images of mitochondria in YY2‐overexpressed, DRP1‐overexpressed HCC‐LM3 stem‐like tumor spheres. Representative images (scale bars: 200 nm) and quantification results (right; *n* = 30) are shown. D) OCR of YY2‐overexpressed, DRP1‐overexpressed HCC‐LM3 stem‐like tumor spheres (*n* = 3). E) Δ*Ψ*_m_ in YY2‐overexpressed, DRP1‐overexpressed HCC‐LM3 stem‐like tumor spheres, as determined using MitoTracker Red/MitoTracker Green. Representative images (scale bars: 10 µm) and quantification results (right; *n* = 10) are shown. F) Δ*Ψ*_m_ in YY2‐overexpressed, DRP1‐overexpressed HCC‐LM3 stem‐like tumor spheres, as determined using JC‐1 staining and flow cytometry n = 3). G) Δ*Ψ*_m_ in DRP1 knock‐down HCC‐LM3^YY2KO^ stem‐like tumor spheres, as determined using MitoTracker Red/MitoTracker Green Representative images (scale bars: 10 µm) and quantification results (right; *n* = 10) are shown. H) Δ*Ψ*_m_ in YY2‐overexpressed, DRP1‐overexpressed HCC‐LM3 stem‐like tumor spheres, as determined using JC‐1 staining and flow cytometry (*n* = 3). Cells transfected with pcCon or wild‐type cells transfected with shCon were used as controls. Quantification data are shown as mean ± SD. *p* values were calculated using two‐tailed unpaired Student's *t*‐test. pcCon: pcEF9‐Puro; FCCP: carbonyl cyanide‐p‐trifluoromethoxyphenylhydrazone. ^**^ *p* < 0.01.

To elucidate the role of DRP1 in YY2‐mediated regulation of mitochondrial fission in CSCs, we constructed a DRP1‐overexpressing vector (Figure S11A, Supporting Information), as well as two short hairpin RNA (shRNA) expression vectors targeting different sites on DRP1. We chose shDRP1‐2 (referred to as shDRP1 hereafter), which exerted a better suppressive effect, for further experiments (Figure S11B,C, Supporting Information). DRP1 overexpression restored the colocalization of DRP1 with DsRed‐Tom20 in YY2‐overexpressed HCC‐LM3 cells obtained from stem‐like tumor spheres (**Figure** **6**A); whereas DRP1 silencing abolished the increase of its level in HCC‐LM3^YY2KO^ cells obtained from stem‐like tumor spheres (Figure 6B).

Furthermore, DRP1 overexpression prevented alterations in mitochondrial morphology observed upon YY2 overexpression (Figure 6C), and partially restored the OCR in stem‐like tumor spheres formed by YY2‐overexpressed HCC‐LM3 cells (Figure 6D). Moreover, Δ*Ψ*_m_ in stem‐like tumor spheres formed by YY2‐overexpressed HCC‐LM3 cells was restored by DRP1 overexpression (Figure 6E,F), while DRP1 silencing abolished the increase of Δ*Ψ*_m_ in stem‐like tumor spheres formed by HCC‐LM3^YY2KO^ cells (Figure 6G,H). Taken together, these results clearly demonstrate that DRP1 is critical for YY2‐mediated regulation of mitochondrial fission.

### 2.7 YY2/DRP1 Axis is Crucial for CSC Asymmetric Division

To examine the role of the YY2/DRP1 axis in the asymmetric division of CSCs, we first altered DRP1 expression and characterized the ensuing liver CSCs. DRP1 silencing reduced the size and number of tumor spheres formed by HCC‐LM3 cells, as well as the frequency of liver CSCs (Figure S12A,B, Supporting Information); whereas DRP1 overexpression enhanced the above values (Figure S12C,D, Supporting Information). Furthermore, using TCGA dataset of HCC patients at various stages, we found a positive correlation between DRP1 and disease progression (Figure S13A, Supporting Information). Accordingly, low DRP1 levels implied significantly higher overall survival than high DRP1 levels (Figure S13B, Supporting Information). These findings contrasted those obtained with YY2, thus, together with abovementioned results showing YY2 negative regulation on DRP1 transcription, pointing to the involvement of DRP1 in YY2‐mediated regulation of disease progression and CSC asymmetric division.

**Figure 7:**
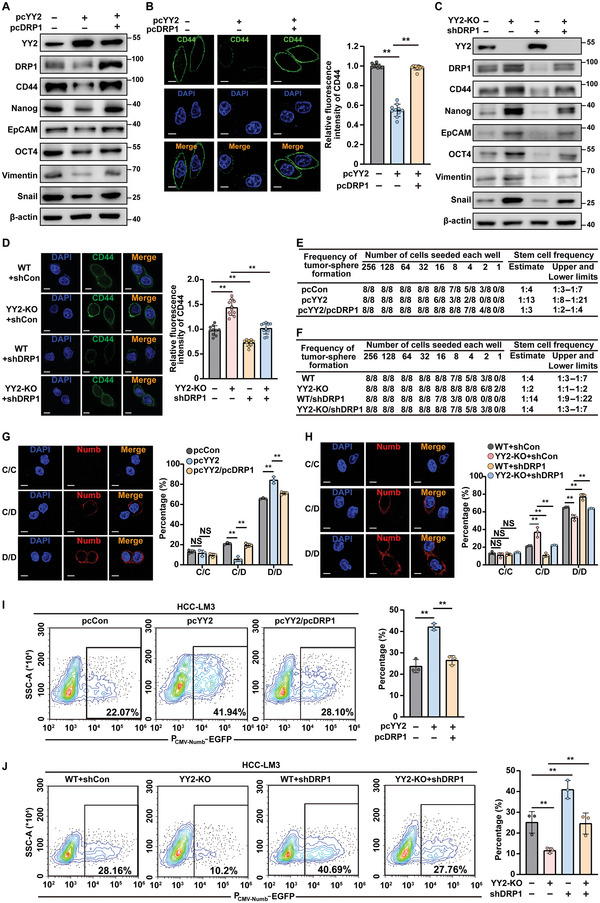
YY2/DRP1 axis is crucial for CSC asymmetric division. A) Protein levels of CSC markers in YY2‐overexpressed, DRP1‐overexpressed HCC‐LM3 cells, as determined using western blotting. B) CD44 fluorescence intensity in YY2‐overexpressed, DRP1‐overexpressed HCC‐LM3 cells. Representative images (scale bars: 10 µm) and quantification results (*n* = 10) are shown. C) Protein levels of CSC markers in DRP1 knock‐down HCC‐LM3^YY2KO^ cells, as determined using western blotting. D) CD44 fluorescence intensity in DRP1 knock‐down HCC‐LM3^YY2KO^ cells. Representative images (scale bars: 10 µm) and quantification results (*n* = 10) are shown. E,F) CSC frequency in YY2‐overexpressed, DRP1‐overexpressed HCC‐LM3 cells (E) and DRP1 knock‐down HCC‐LM3^YY2KO^ cells (F), as determined using in vitro LDA. G,H) Sphere cell division types in YY2‐overexpressed, DRP1‐overexpressed HCC‐LM3 cells (G), and DRP1 knock‐down HCC‐LM3^YY2KO^ cells (H). Immunofluorescence of Numb (red) and DAPI (blue) representing three division types: CSC/CSC (C/C; Numb^−^/Numb^−^), CCSC/non‐CSC (C/D; Numb^−^/Numb^+^), and non‐CSC/non‐CSC (D/D; Numb^+^/Numb^+^). Representative images (left; scale bars: 10 µm) and quantification results from three independent experiments (right, each dot represents 24 to 32‐pairs of daughter cells) are shown. I,J) Percentages of EGFP‐positive cells in YY2‐overexpressed, DRP1‐overexpressed HCC‐LM3 cells (I) and DRP1 knock‐down HCC‐LM3^YY2KO^ cells (J) transfected with P_CMV‐Numb_‐EGFP vector, as analyzed using flow cytometry (*n* = 3). *β*‐actin was used as western blotting loading control. Cells transfected with pcCon or wild‐type cells transfected with shCon were used as controls. Quantification data are shown as mean ± SD. *p* values were calculated using two‐tailed unpaired Student's *t*‐test. pcCon: pcEF9‐Puro; ^**^ *p* < 0.01; ns: not significant.

To better understand the role of DRP1 and YY2 in the asymmetric division of CSCs, we overexpressed both YY2 and DRP1 in HCC‐LM3 cells. DRP1 overexpression restored the levels of CSC markers (**Figure** **7**A,B), as well as the number of tumor spheres suppressed by YY2 overexpression (Figure S14A, Supporting Information). In contrast, DRP1 silencing abrogated the increase in CSC markers and the number of tumor spheres in HCC‐LM3^YY2KO^ cells (Figure 7C,D; Figure S14B, Supporting Information). Furthermore, DRP1 overexpression increased the frequency of liver CSCs in YY2‐overexpressed cells from 1:13 to 1:3 (Figure 7E); whereas DRP1 silencing reduced it from 1:2 to 1:4 in HCC‐LM3^YY2KO^ cells (Figure 7F). These results indicated that the negative regulation of DRP1 by YY2 was crucial for downregulating HCC stemness.

Furthermore, while YY2 overexpression suppressed liver CSC asymmetric division and promoted its symmetric division into two non‐CSC cells in stem‐like tumor spheres formed by HCC‐LM3 cells, DRP1 overexpression restored asymmetric division and blocked the symmetric one (Figure 7G). In agreement with these results, DRP1 silencing counteracted the increased number of asymmetric divisions in HCC‐LM3^YY2KO^ cells and restored the proportion of symmetric divisions into non‐CSC cells (Figure 7H). This finding implied a critical role of DRP1 in the YY2‐dependent negative regulation of CSC asymmetric division. Moreover, although YY2 overexpression increased the percentage of EGFP‐positive cells driven by CMV‐Numb or CMV‐Albumin promoters in HCC‐LM3 cells, DRP1 overexpression reversed these effects (Figure 7I; Figure S15A, Supporting Information). In contrast, DRP1 silencing enhanced the proportion of EGFP‐positive cells driven by the CMV‐Numb or CMV‐Albumin promoters in HCC‐LM3^YY2KO^ cells (Figure 7J; Figure S15B, Supporting Information). Taken together, these results demonstrate that YY2 suppresses CSC asymmetric division and enhances CSC differentiation by downregulating DRP1 transcription.

### 2.8 Regulation of Mitochondrial Fission by the YY2/DRP1 Axis is Crucial for CSC Homeostasis and Tumorigenesis

**Figure 8:**
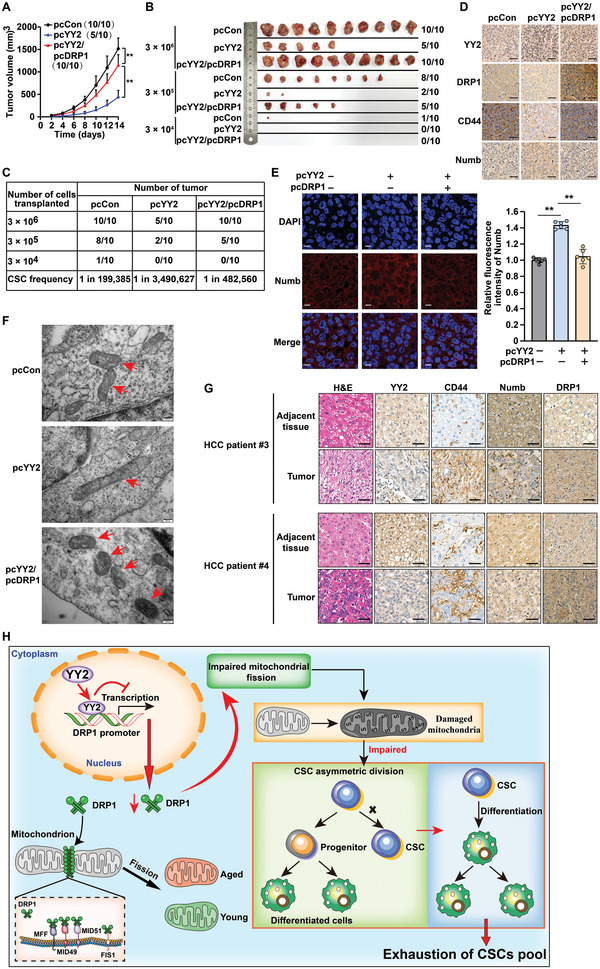
YY2/DRP1 is crucial for mitochondrial fission‐regulated CSC homeostasis and HCC tumorigenic potential. A–C) Tumor‐initiating potential of YY2‐overexpressed HCC‐LM3 cells, as examined by in vivo LDA using xenograft experiment. Tumor volume (A), morphological images (B), and CSC frequencies (C) are shown. Ratio of the number of mice with tumor to the number of total mice transplanted with indicated cells is shown. D) Immunohistochemical staining of YY2, DRP1, CD44, and Numb in the tissue section of xenografted tumors formed by YY2‐overexpressed, DRP1‐overexpressed HCC‐LM3 cells (scale bars: 50 µm). E) Numb fluorescence intensity in the xenografted tumor lesions formed by YY2‐overexpressed, DRP1‐overexpressed HCC‐LM3 cells, as examined using immunofluorescence staining. Representative images (left; scale bars: 10 µm) and quantification results (right; *n* = 6) are shown. F) Transmission electron microscopy images of mitochondria in the xenografted tumor lesions formed by YY2‐overexpressed, DRP1‐overexpressed HCC‐LM3 cells (scale bars: 200 nm). G) YY2, CD44, Numb, and DRP1 expression levels in clinical HCC tissues and the corresponding normal adjacent tissue, as analyzed using immunohistochemical staining. Scale bars: 50 µm. H) Schematic diagram showing YY2/DRP1 axis regulation on CSC pool maintenance through mitochondrial fission‐mediated CSC asymmetric division. Tumor lesions formed by HCC‐LM3 cells transfected with pcCon were used as controls. Quantification data are shown as mean ± SD. *p* values were calculated using two‐tailed unpaired Student's *t*‐test. For xenograft experiments, *p* values were calculated using one‐way ANOVA. pcCon: pcEF9‐Puro; ^**^ *p* < 0.01.

To elucidate the pathological function of the YY2/DRP1 pathway in vivo, especially in regulating tumor‐initiating capacity, we established stable HCC‐LM3/pcCon, HCC‐LM3/pcYY2, and HCC‐LM3/pcYY2/pcDRP1 cell lines (Figure S16A, Supporting Information), and transplanted them subcutaneously into BALB/c‐nu/nu mice. YY2 overexpression significantly reduced the tumorigenic potential of HCC‐LM3 cells, whereas DRP1 overexpression restored it (**Figure** **8**A; Figure S16B, Supporting Information). An in vivo LDA revealed that DRP1 overexpression robustly restored liver CSC frequency, which decreased significantly in tumors formed by YY2‐overexpressed HCC‐LM3 cells (Figure 8B,C). Immunohistochemistry showed that DRP1 and CD44 were downregulated in the xenografted tumors formed by YY2‐overexpressed HCC‐LM3 cells, in which Numb was upregulated; whereas DRP1 overexpression restored CD44 levels and resuppressed Numb (Figure 8D; Figure S16C–F, Supporting Information). Similar results were obtained from immunofluorescence staining for Numb (Figure 8E). These findings clearly showed that the negative regulation of DRP1 by YY2 suppressed HCC tumor‐initiating capacity by disrupting CSC homeostasis and promoting CSC differentiation, thereby depleting the CSC pool.

Meanwhile, transmission electron microscopy revealed once again distorted “‘spaghetti‐like”’ mitochondria in the tumor lesions formed by YY2‐overexpressed HCC‐LM3 cells; whereas mitochondria in the lesions formed by HCC‐LM3/pcYY2/pcDRP1 cells were more fragmented and less tubular (Figure 8F; Figure S16G, Supporting Information). This result indicated that regulation of mitochondrial fission by the YY2/DRP1 axis was crucial for HCC tumor‐initiating capacity.

Finally, we assessed the expression of YY2, CD44, Numb, and DRP1 in two HCC clinical samples. As shown in Figure 8G, compared to the corresponding adjacent tissue, YY2 was downregulated in tumor lesions; whereas DRP1 expression was significantly increased. Furthermore, the CSC marker CD44 was also upregulated in tumor lesions, while Numb showed a positive correlation with YY2. These results were in agreement with our cellular and animal experimental data pointing to YY2 as a DRP1 transcriptional suppressor, as well as with the negative correlation between the YY2/DRP1 axis and CSC asymmetric division.

Overall, our results clearly show that YY2 could suppress the tumor‐initiating capacity of HCC cells by inhibiting DRP1‐mediated CSC mitochondrial fission, thereby downregulating CSC asymmetric division and subsequently, exhausting the CSC pool (Figure 8H).

## 3 Discussion

CSCs play a pivotal role not only in tumor initiation, but also in therapeutic resistance, metastasis, and recurrence in multiple types of cancers.^[^ ^32^ ^]^ Asymmetric division, whereby a CSC divides into a cell that maintains its stem cell characteristics and another more differentiated transit‐amplifying cell, is crucial for maintaining the pool of CSCs and their tumor‐initiating potential.^[^ ^6^, ^33^ ^]^ Herein, we identified YY2, an activating or inhibitory zinc‐finger transcription factor belonging to the yin‐yang family,^[^ ^34^ ^]^ as a novel negative regulator of CSCs. We report that YY2 impairs asymmetric stem cell division and promotes the generation of more differentiated progeny from liver CSCs, thereby suppressing the tumor‐initiating capacity and tumorigenesis of CSCs. Considering that YY2 is downregulated in clinical HCC lesions as well as in other cancers,^[^ ^23^, ^35^, ^36^ ^]^ and that YY2 downregulation correlates with disease progression and poor prognosis in liver cancer patients, these results suggest a novel YY2‐dependent regulatory mechanism acting on CSCs, along with new therapeutic opportunities for pharmacological targeting CSCs asymmetric division.

Homeostasis of mitochondrial dynamics, which depends on the balance between mitochondrial fission and fusion, plays an important role in maintaining survival and self‐renewal of both stem cells and CSCs.^[^ ^15^, ^17^, ^37^, ^38^, ^39^ ^]^ Mitochondrial fission, whereby mitochondrion divide asymmetrically to produce a healthy and a dysfunctional mitochondrion, is crucial for removing dysfunctional organelles by autophagy while ensuring a pool of healthy mitochondria.^[^ ^30^ ^]^ Furthermore, it enables asymmetric partitioning of healthy and dysfunctional mitochondria to the two progenies of CSCs, thereby promoting CSC asymmetric division.^[^ ^16^, ^17^, ^40^ ^]^ Previous studies have reported that the rate of mitochondrial fission is higher in CSCs.^[^ ^41^ ^]^ Impaired mitochondrial fission disrupts the asymmetric division and self‐renewal capacity of CSCs, leading to CSC differentiation and senescence, which eventually limits the CSC pool and tumor‐initiating capacity.^[^ ^17^, ^38^, ^40^ ^]^ Therefore, identifying key factors that target CSC‐specific mitochondrial fission may be of great clinical value. Our study shows that YY2 binds directly to the DRP1 promoter and acts as a transcriptional suppressor, thereby impairing mitochondrial fission and the asymmetric division of CSCs. Given that DRP1 is a large dynamin‐related GTPase whose recruitment to the mitochondrial surface is indispensable for mitochondrial fission, our results reveal a novel function of YY2, as well as a crucial novel regulatory pathway for mitochondrial fission. Furthermore, abnormal mitochondrial fission is associated with various pathological conditions, including neurodegenerative disorders, cardiovascular diseases, obesity, and embryonic lethality.^[^ ^15^, ^17^, ^39^ ^]^ Hence, although further studies are needed, the YY2/DRP1 axis could be implicated in multiple biological processes and pathological conditions.

CSCs are generally quiescent and less prone to proliferation than bulk tumor cells, making them more resistant to chemotherapy and radiation. Since most of these treatments trigger tumor cell apoptosis by inducing DNA damage, which can be detected by cell cycle checkpoints, but not in the G_0_ phase.^[^ ^14^, ^42^, ^43^, ^44^ ^]^ Therefore, exhausting the CSC pool by promoting the differentiation of CSCs has attracted attention as a potential strategy for improving tumor cell sensitivity to chemotherapeutic agents and radiotherapy in clinical treatment, as well as for preventing tumor metastasis and recurrence.^[^ ^45^ ^]^ Our results demonstrate that YY2 overexpression promotes the differentiation of liver CSCs into more mature HCC cells, thus depleting the liver CSC pool. Given the enhanced sensitivity toward chemotherapeutic agents of the resulting tumor cells, YY2 could be targeted in antitumor treatments.

In summary, while further investigations are needed to confirm the role of YY2/DRP1 axis in regulating CSC in tumors other than HCC, in this study we identified YY2 as a novel regulator of CSC asymmetric division, and linked it with DRP1‐mediated mitochondrial fission. Furthermore, we provide strong evidence that YY2 overexpression, which promotes CSC differentiation and exhaustion of the CSC pool, could sensitize CSCs to DNA damage inducers. Hence, our findings not only highlight an unprecedented relationship between YY2, mitochondrial fission, and CSC self‐renewal, but also provide novel insights into the regulatory mechanism of mitochondrial fission in CSCs, as well as the molecular pathway underlying the tumor suppressive effect of YY2. Finally, our study points to YY2 as a novel therapeutic target for liver cancer.

## 4 Experimental Section

### Plasmids and Constructs

YY2 overexpression vector and YY2 shRNA expression vectors targeting two different sites of YY2 were constructed as described previously.^[^ ^23^ ^]^ For shRNA expression vectors targeting DRP1, target sites were designed using the algorithm and method previously reported.^[^ ^46^, ^47^ ^]^ The target sequences were as follows: shYY2‐1: 5“‐GCA TCA ACA TCA ACA TCA A‐3”; shYY2‐2: 5“‐ACA TCA ACA TCA ACC CAG A‐3”; shDRP1‐1: 5'‐ GTA TGA ACG ACT ATA TTA T‐5'; and shDRP1‐2: 3“‐GGT CCA TGT TTC ACA AGA A‐5”. Cancer‐associated mutant YY2 overexpression vectors (P232L, G260A, and E212K), were constructed using Site‐directed Mutagenesis Kit (Beyotime Biotechnology, Shanghai, China).

For DRP1 overexpression vector, the coding region of human DRP1 was amplified using the Takara Ex Taq Kit (Takara Bio, Dalian, China) from human cDNA obtained by reverse‐transcribing total RNA extracted from HCC‐LM3 cells using the PrimeScript RT Reagent Kit with gDNA Eraser (Takara Bio). The amplicon was inserted into the BamHI and EcoRI sites of pcEF9‐Puro vector.^[^ ^48^ ^]^ For DsRed2‐Tom20 fusion expression plasmid, the coding region of human Tom20 was inserted into the XhoI and EcoRI sites of pDsRed2‐C1 vector (Invitrogen Life Technologies, Carlsbad, CA).

For reporter vectors bringing different regions of DRP1 promoter (Refseq No. NC_ 000012.12; DRP1‐Luc‐1 with the ‐2071 to +273 region; DRP1‐Luc‐2 with the −1509 to +273 region, DRP1‐Luc‐3 with the −1127 to +273 region, DRP1‐Luc‐4 with the −646 to +273 region, and DRP1‐Luc‐5 with the ‐646 to ‐126 region), a modified pGL4.13 vector is constructed by inserting MluI, NdeI, EcoRI, and SmaI sites between the HindIII and ApaI sites in the multi‐cloning sites of pGL4.13 vector (Promega, Madison, WI). Human genome DNA extracted from HCC‐LM3 hepatocellular carcinoma (HCC) cells using Genomic DNA Kit (Tiangen Biotech, Beijing, China) was used as template for amplifying corresponding promoter regions using Takara PrimeSTAR Max DNA Polymerase (Takara Bio). DRP1 luciferase reporter vector with mutated YY2 binding site (DRP1‐Luc^mut^) was constructed from DRP1‐Luc‐1 by mutating the corresponding site using Site‐directed Mutagenesis Kit (Beyotime Biotechnology. Numb and Albumin EGFP reporter vectors were constructed by inserting human Numb promoter (Refseq No. NC_ 000014.9) or human Albumin promoter (Refseq No. NC_ 000004.12) obtained by amplifying human genome DNA as described above into the XhoI and EcoRI sites of pEGFP‐N1 vector (Invitrogen Life Technologies, Carlsbad, CA).

### Cell Lines and Cell Culture

HCC‐LM3, MHCC‐97H, and HepG2 hepatocarcinoma (HCC) cells were purchased from the Cell Bank of Chinese Academy of Sciences (Shanghai, China), and cultured in Dulbecco's modified Eagle's medium (DMEM; Gibco, Life Technologies, Grand Island, NY) with 10% fetal bovine serum (FBS; Biological Industries, Beith Haemek, Israel) and 1% penicillin–streptomycin. Cell lines were verified using short‐tandem repeat profiling method, and were tested periodically for mycoplasma contamination by using Mycoplasma Detection Kit‐QuickTest (Biotool, Houston, TX). For gene‐silencing and gene‐overexpression experiments, cells were seeded in 6‐well plate and transfected with 2 *µ*g of indicated vector. Twenty‐four hours after transfection, transfected cells were selected using 1 µg mL^−1^ puromycin for 36 h. For double silencing and double overexpression experiments, cells were transfected with 1 *µ*g of each indicated vectors, and subjected to puromycin selection to eliminate untransfected cells. YY2 knock‐out cells were established using CRISPR/Cas9 method. Briefly, cells were transfected with vectors targeting YY2 (HCP301990‐CG04‐3‐10‐a, target site: 5″‐GAT GGC AAT TGG ATC TACGG‐3″; HCP301990‐CG04‐3‐10‐b, target site: 3″‐TAG CCC GTG TTC GTGAAG AG‐5″; HCP301990‐CG04‐3‐10‐c, target site: 3″‐TCC GTC GGA ATGTCC TCC AT‐5″; Gene Copoiea, Rockville, MD). Twenty‐four hours later, neomycin selection (600 ng mL^−1^) was performed for 10 days to eliminate untransfected cells. Cell line was then established from a single clone. Deletion of nucleotides located in +97 to +195 region (98 bp) of YY2 coding sequence was confirmed by sequencing. All transfections were performed using Lipofectamine 2000 (Invitrogen Life Technologies) according to the manufacturer's instruction.

### Clinical Human HCC Specimen

Human HCC specimens were obtained from HCC patients undergoing surgery at Chongqing University Cancer Hospital (Chongqing, China), snap‐frozen in liquid nitrogen, and stored in Biological Specimen Bank of Chongqing University Cancer Hospital. Patients did not receive chemotherapy, radiotherapy, or other adjuvant therapies prior to the surgery. Prior patient's written informed consents were obtained. The experiments were approved by the Institutional Research Ethics Committee of Chongqing University Cancer Hospital (Permit No. CZLS2021292‐A), and conducted in accordance with the Declaration of Helsinki.

### Animal Experiments

For the in vivo tumor study, BALB/c‐nu/nu mice (male, body weight: 18–22 g, 6 weeks old) were purchased from the Chongqing Medical University (Chongqing, China). Animal studies were conducted in the Chongqing University Cancer Hospital, and approved by the Laboratory Animal Welfare and Ethics Committee of Chongqing University Cancer Hospital. All animal experiments conformed to the approved guidelines of the Animal Care and Use Committee of the Chongqing University Cancer Hospital (Permit No. SYXK‐2021‐0001). All efforts to minimize suffering were made.

For xenograft experiments, BALB/c‐nu/nu mice were randomly divided and subjected to in vivo LDA by subcutaneously injecting indicated numbers of cells. Tumor size (V) was evaluated by a caliper every 2 days using the following equation: V = a × b^2^/2; whereas a and b were the major and minor axes of the tumor, respectively. The investigator was blinded to the group allocation during the assessment. CSC frequencies were analyzed using L‐Calc v1.1 (Stem Cells Technologies, Inc., Vancouver, Canada) based on a Poisson distribution.^[^ ^49^ ^]^

### Spheroid Formation Assays and in Vitro LDA

HCC cells were seeded in 6‐ or 96‐well ultra‐low attachment plates (Corning Life Sciences, Corning, NY, USA) and cultured for 7 days in DMEM/F12 medium (Gibco) enriched with B27 and N2 supplements, 20 ng mL^−1^ epidermal growth factor, and 20 ng mL^−1^ basic fibroblast growth factor (Invitrogen Life Technologies). The generated spheroids were counted under a microscope.

For the in vitro LDA, the indicated numbers of HCC cells were seeded in 96‐well ultra‐low attachment plates and cultured as described above for 7 days. CSC frequencies were analyzed using L‐Calc v1.1 (Stem Cell Technologies) based on a Poisson distribution.^[^ ^49^ ^]^

### Asymmetric Division Assay

For assessing CSC asymmetric division, the cells obtained from stem‐like tumor spheres into the confocal dish were reseeded. Twelve hours later, cells were synchronized at M phase by treating them with nocodazole (final concentration: 100 ng mL^−1^) for 12 h. Cells were washed with PBS, and cultured with normal medium for 1 h before being fixed with 4% paraformaldehyde for 30 min at room temperature, and permeabilized with PBS containing 0.1% Triton X‐100 for 5 min. After blocking with 1% bovine serum albumin for 1 h, cells were incubated with Numb antibodies for 2 h followed by incubation with fluorescent secondary antibodies for 1 h. Nuclei were stained with DAPI (Beyotime Biotechnology) for 15 min. Images were taken and analyzed with laser scanning confocal microscopy (Leica Microsystems TCS SP5).

### RNA Sequencing and Data Analysis

Following RNA extraction, RNA‐Seq analysis was performed by Novogene (Beijing, China) using an Illumina HiSeq 2500 instrument (Illumina, San Diego, CA) with three repetitions per group. Raw reads were preprocessed by filtering out rRNA reads, sequencing adapters, short‐fragment reads, and other low‐quality reads. TopHat v2.1.0 was used to map the clean reads to the human reference genome ensemble GRCh38 (hg38) with two mismatches. After genome mapping, Cufflinks v2.1.1 was run with reference annotations to generate fragments per kilobase per million mapped reads (FPKM) values for known gene models. Differentially expressed genes were identified using Cuffdiff software. The significance threshold for differentially expressed genes in multiple tests was set based on a false discovery rate ≤ 0.05. The fold‐changes were estimated according to FPKM values in each sample.

### Transmission Electron Microscopic Analysis

Samples were fixed with 2.5% glutaraldehyde, washed with 0.1 m phosphate buffer (pH 7.4), and post‐fixed with 1% osmium 0.1 m phosphate buffer. Samples were then dehydrated in increasing concentrations of ethanol, infiltrated, and embedded in SPI‐Pon812 before being polymerized in a 60°C oven for 48 h. Ultrathin sections were cut using a Leica Ultracut microtome (Leica, Heidelberg, Germany) and loaded on formvar and carbon‐coated copper grids. Grids were photographed using transmission electron microscope (HITACHI HT7700, Tokyo, Japan).

### Mitochondrial Membrane Potential Analysis

Cells were seeded in a 6‐well ultra‐low attachment culture plate (2000 cells per well) and cultured for 7 days. Mitochondria were stained using MitoTracker Red and MitoTracker Green (Invitrogen Life Technologies; cat# M22425 and M7514, respectively) according to the manufacturer's instructions. Nuclei were stained with Hoechst. Fluorescence images of tumor‐sphere cells were obtained using laser scanning confocal microscopy (Leica Microsystems TCS SP5).

### OCR Measurement

For OCR measurement, cells were plated in XF8 cell‐culture microplate (Agilent, Santa Clara, CA; 1 × 10^4^ cells per well) and cultured overnight. The medium was then replaced with the XF assay medium containing 1 mM pyruvate (Agilent), 2 mM glutamine (Agilent), and 10 mM glucose (Agilent). OCR was measured using Seahorse Analyzer XF8 (Agilent) by sequential addition of XF Cell Mito Stress Kit (Agilent) containing oligomycin (final concentration: 2 µM), FCCP (final concentration: 1 µM), and Rotenone/antimycin A (final concentration: 0.5 µM) according to the manufacturer's instructions. Data were normalized to cell number and were plotted as mean ± SD.

### Anchorage‐Independent Colony‐Forming Assay (Soft Agar Assay)

Agarose (1.2%, #A9045; Sigma Aldrich, St. Louis, MO, USA) and 2× DMEM (Gibco) were mixed 1:1 and plated in a 6‐well plate (50 mL per well). Once the bottom layer had gelled, 100 cells suspended in 2 mL medium with 0.35% agarose were plated on top to create a soft agar layer. After solidification, 2 mL DMEM containing 10% FBS (Biological Industries) and 1% penicillin–streptomycin was added on the agar. The culture medium was replaced with fresh medium every 2 days. Colonies were counted on day 14. Images were taken using an Olympus IX71 microscope (Olympus, Tokyo, Japan).

### RNA Extraction and Quantitative Reverse Transcription‐PCR (qRT‐PCR)

Total RNA (1 *µ*g) was extracted using Trizol (Invitrogen Life Technologies) according to the manufacturer's instruction, then reverse‐transcribed into cDNA using PrimeScript Reagent Kit with gDNA Eraser (Takara Bio). qRT‐PCR was performed using SYBR Premix ExTaq (Takara Bio). The sequences of the primers used are listed in Table S1 (Supporting Information). *β*‐actin was used to normalize sample amplification. The results were shown as relative to the expression level in the corresponding controls, which were assumed as 1.

### Flow Cytometry

Cells were prepared as described above, resuspended in cold flow cytometry buffer, and stained with corresponding antibodies or JC‐1 (ENZ‐52305, ENZO, Life Science, NY, USA). After being washed, resuspended in cold flow cytometry buffer, and passed through a cell strainer, cells were subjected to flow cytometry using CytoFLEX flow cytometer (Beckman Coulter, Brea, CA). Cells expressing fluorescence reporter genes were subjected directly to flow cytometry after being resuspended in cold flow cytometry buffer and passed through cell strainer. The antibodies used were listed in Table S2 (Supporting Information).

### Western Blotting

Total cells were lysed with RIPA lysis buffer with protease inhibitor and phosphatase inhibitor cocktail (complete cocktail; Roche Applied Science, Mannheim, Germany). Equal amounts of the sample proteins were electrophoresed on sodium dodecyl sulphate polyacrylamide gel before being transferred to a polyvinylidene fluoride membrane with 0.45 µm pores (Millipore, Billerica, MA). Membranes were then incubated with first antibodies followed by second antibodies. Antibodies used are listed in Table S2 (Supporting Information), and immunoblotting with anti‐*β*‐actin antibody was conducted to ensure equal protein loading. Signals were measured using Super Signal West Femto Maximum Sensitivity Substrate detection system (Thermo Fisher Scientific, Waltham, MA). For xenografted tissues and clinical HCC samples, tissues were frozen in liquid nitrogen and ground before being lysed with RIPA lysis buffer with protease inhibitor and phosphatase inhibitor cocktail (complete cocktail; Roche Applied Science). Western blotting was performed as described above, and immunoblotting with anti‐GAPDH antibody was conducted to ensure equal protein loading for samples from xenografted tissues. Images of uncropped blots are shown in Figure S17 (Supporting Information) (A to M).

### ChIP Assay

Chromatin was immunoprecipitated using the ChIP Assay Kit (Beyotime Biotechnology) according to the manufacturer's instructions. Briefly, cells were lysed and chromatins were immunoprecipitated using protein A+G Agarose/salmon sperm DNA and anti‐YY2 antibody, anti‐H3 antibody, or normal mouse IgG, de‐crosslinked for 4 h at 65°C, and treated with 0.5 M EDTA, 1 M Tris (pH 6.5), and 20 mg mL^−1^ proteinase K. Immunoprecipitated chromatin was then subjected to PCR by using PrimeSTAR Max (Takara Bio). Primer sequences used for amplifying the DRP1 promoter region with the predicted YY2 binding site were: 5'‐ CTCCTCTCCACCTCCCTCG‐3' (forward); and 5'‐ CTCACCTGCGTTCCCACTAC‐3' (reverse).

### Dual Luciferase Reporter Assay

Cells were seeded into 24‐well plates (5 × 10^4^ cells per well). Twenty‐four hours later, cells were co‐transfected with indicated overexpression vectors, reporter vector and Renilla luciferase expression vector (pRL‐SV40, Promega) as internal control. Luciferase activities were measured with Dual Luciferase Assay System (Promega) 48 h after co‐transfection. Firefly luciferase's activities were normalized to the corresponding Renilla luciferase's activities. The results were shown as relative to the expression level in the corresponding controls, which were assumed as 1.

### Immunofluorescence Staining

Cells were seeded in 3.5‐cm confocal dishes (Thermo Fisher Scientific; 3 × 10^4^ cell per dish), fixed with 4% paraformaldehyde for 30 min at room temperature, and then permeabilized with PBS containing 0.1% Triton X‐100 for 5 min. After blocking with 1% bovine serum albumin for 1 h, cells were incubated with primary antibodies for 2 h followed by incubation with fluorescent secondary antibodies for 1 h. Nuclei were stained with DAPI (Beyotime Biotechnology) for 15 min. Images were taken and analyzed with laser scanning confocal microscopy (Leica Microsystems TCS SP5). Antibodies used were listed in Table S2 (Supporting Information).

### Immunohistochemistry (IHC) and Hematoxylin‐Eosin (H&E) Staining

Fresh human HCC tissues, normal adjacent tissues, and xenografted tumors were fixed using 4% paraformaldehyde for overnight prior to being embedded in paraffin and sectioned at 4 µm thickness using a cryostat. Sections were then dewaxed using xylene and rehydrated prior being incubated with primary antibodies for 1 h, followed by incubation with corresponding secondary antibodies conjugated with horse‐radish peroxidase for 1 h. Visualization was performed using a DAB Kit (DAKO, Beijing, China) under microscope. Nuclei were then counterstained with hematoxylin (Beyotime Biotechnology), followed by dehydration and coverslip mounting. The antibodies used were listed in Table S2 (Supporting Information). Images were taken using Pannoramic Midi (3DHistech, Budapest, Hungary).

For H&E staining, paraffin sections from human HCC tissues and normal adjacent tissues, as well as from mice subcutaneous tumors generated in xenograft experiment (4 µm thickness) were fixed in 10% formalin and washed with 60% propylene glycerol. Samples were then stained with 0.5% hematoxylin‐eosin (Sangon Bio, Shanghai, China) for 3 min followed by dehydration and coverslip mounting. Images were taken using Pannoramic Midi (3DHistech).

### Migration and Invasion Assay

For migration assay, cells were prepared as described above, seeded in the inner plate of a 24‐well plate transwell chamber (Corning Life Sciences), and cultured in 100 mL culture medium supplemented with 10% FBS (Biological Industries). For invasion assay, the inner chamber was coated with an extra‐layer of Matrigel (Corning Life Sciences). Twenty‐four hours later, the medium was replaced with serum‐free medium. The outer chamber was filled with 700 mL culture media supplemented with 10% FBS and further cultured for 24 h. Cells were fixed with 4% paraformaldehyde and stained with crystal violet. Non‐migrated or non‐invaded cells remained on the inner plate were scrapped‐off using a cotton swab. Images of the migrated or invaded cells were taken with Olympus IX71 (Olympus).

### Cell Viability Assay and Calculation of IC_50_

Cells were prepared as described above, seeded into 96‐well plates (4 × 10^3^ cells per well), and treated with the indicated dose of cisplatin. Cell numbers were measured by colorimetric assay with 3‐(4,5‐dimethylthiazol‐2‐yl)‐5‐(3‐carboxymethoxyphenyl)‐2‐(4‐sulfophenyl)‐2H‐tetrazolium (MTS, Promega) at the indicated time points. IC_50_ was calculated based on the results of cell viability using CompuSyn® (https://www.combosyn.com; Combosyn Inc. Paramus, NJ).

### Statistical Analysis

For qRT‐PCR, *β*‐actin was used for normalization. For dual luciferase reporter assay, firefly luciferase activities were normalized with those of Renilla. Quantification results were presented as the mean ± SD (n = 3; unless otherwise indicated). Statistical analysis was performed using two‐tailed unpaired Student's *t*‐test conducted using SPSS Statistics v17.0 (IBM, Chicago, IL). For clinical samples and xenograft experiments, one‐way ANOVA was performed. A value of *p* < 0.05 was considered statistically significant.

## Conflict of Interest

The authors declare no conflict of interest.

## Author Contributions

Conceptualization was done by S.W., V.K., and M.W. Methodology was done by V.K., S.W., M.W., G.S., and M.M. Investigation was done by M.W., U.N., J.L., X.L. R.H., Y.L., J.Z., and W.D. Visualization was done by M.W., U.N., J.L., and X.L. Supervision was done by V.K. and S.W. Writing—original draft was done by V.K., S.W., and M.W. Writing—review and editing was done by V.K. and S.W.

## Supporting information

Supporting Information

## Data Availability

The data that support the findings of this study are available from the corresponding author upon reasonable request.
