# Supplementary material for: YY2‐DRP1 Axis Regulates Mitochondrial Fission and Determines Cancer Stem Cell Asymmetric Division
Source: Adv Sci (Weinh). 2023 Jun 9;10(23):2207349. doi: 10.1002/advs.202207349 (PMC10427375; doi:10.1002/advs.202207349)
Supplement: Supplementary file 1 — Supporting Information [file ADVS-10-2207349-s001.pdf]

ADVANCED SCIENCE
Open Access

Supporting Information

for *Adv. Sci.*, DOI 10.1002/advs.202207349

YY2-DRP1 Axis Regulates Mitochondrial Fission and Determines Cancer Stem Cell Asymmetric Division

Mankun Wei, Uli Nurjanah, Juan Li, Xinxin Luo, Rendy Hosea, Yanjun Li, Jianting Zeng, Wei Duan, Guanbin Song, Makoto Miyagishi, Vivi Kasim and Shourong Wu**

Supplementary Materials for

YY2-DRP1 axis regulates mitochondrial fission and determines cancer stem cell asymmetric division

Mankun Wei, Uli Nurjanah, Juan Li, Xinxin Luo, Rendy Hosea, Yanjun Li, Jianting Zeng, Wei Duan, Guanbin Song, Makoto Miyagishi, Vivi Kasim*, Shourong Wu*

*E-mail: vivikasim@cqu.edu.cn (V.K.)
shourongwu@cqu.edu.cn (S.W.)

This PDF file includes:

Figure S1. YY2 is negatively correlated with disease progression in clinical HCC patients.

Figure S2. *YY2* overexpression suppresses CSC markers expression levels.

Figure S3. YY2 negatively alters liver CSC frequency.

Figure S4. Effect of YY2 alteration on HCC cells migration potential and drug resistance.

Figure S5. YY2 suppresses HCC cell stemness *in vivo*.

Figure S6. YY2 promotes liver CSC differentiation.

Figure S7. YY2 decreased mitochondrial membrane potential ($\Delta\Psi$m).

Figure S8. YY2 negatively regulates $\Delta\Psi_m$ by suppressing mitochondrial fission.

Figure S9. Mitochondrial fission is crucial for YY2 regulation on tumor sphere formation potential.

Figure S10. YY2 regulates *DRP1* transcription.

Figure S11. Efficacies of *DRP1* overexpression vector and shRNA expression vectors targeting *DRP1*.

Figure S12. DRP1 promotes HCC cells stemness.

Figure S13. DRP1 expression level positively correlates with disease progression and poor prognosis in clinical HCC patients.

Figure S14. DRP1 downregulation is crucial for YY2 regulation on stem-like tumor sphere formation potential.

Figure S15. YY2 promotes liver CSC differentiation by suppressing DRP1.

Figure S16. YY2 mediates HCC tumorigenesis potential by regulating DRP1.

Figure S17. Uncropped western blots with the indicated areas of selection in Figs.1, 2, 5, 7 and Supplementary Figs. S2, S4, S5, S6, S10, S11, S16.

Table S1. Primer pairs used for qRT-PCR.

Table S2. Antibodies used for western blotting, immunofluorescence, immunohistochemistry, and ChIP assay.

Supplementary Figure S1

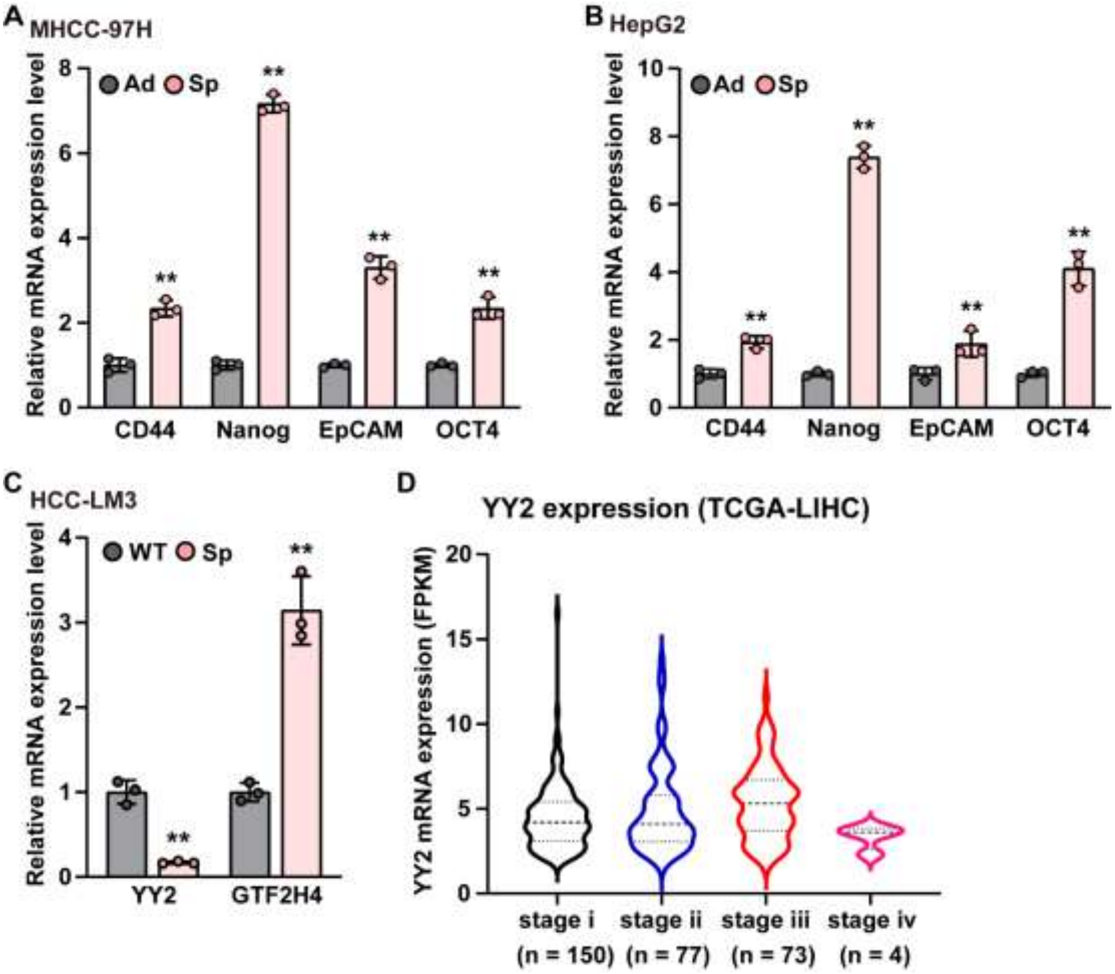

Figure S1. YY2 is negatively correlated with disease progression in clinical HCC patients. (A and B) mRNA expression levels of CSC markers in adherent and stem-like tumor spheres formed by MHCC-97H (A) and HepG2 (B) cells, as determined using qRT-PCR. **(C)** mRNA expression levels of YY2 and GTF2H4 in adherent and stem-like tumor spheres formed by HCC-LM3, as determined using qRT-PCR. **(D)** Correlation between YY2 expression level and HCC disease progression, as analyzed using TCGA dataset. β-actin was used for qRT-PCR normalization. Quantification data are shown as mean ± SD (n = 3). *P* values were calculated using two-tailed unpaired Student's *t*-test. Ad: adherent cells; Sp: stem-like tumor spheres; LIHC: liver hepatocellular carcinoma; $^{**}P < 0.01$.

Supplementary Figure S2

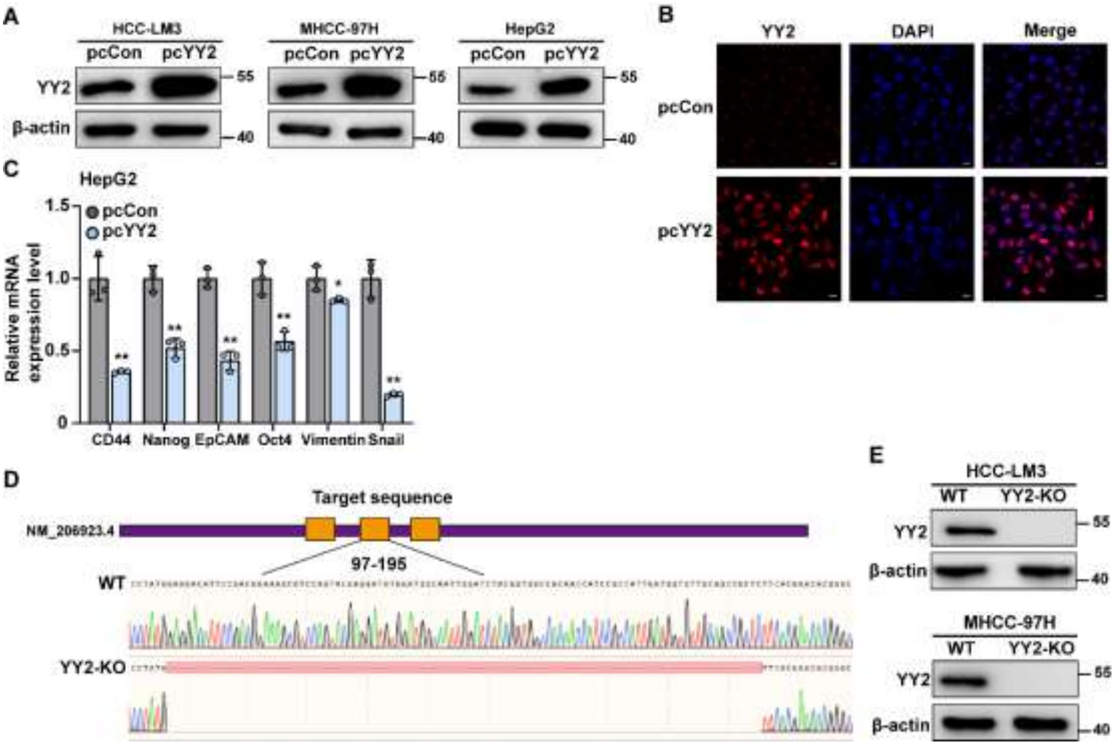

Figure S2. ***YY2*** **overexpression suppresses CSC markers expression levels. (A)** YY2 protein expression level in HCC-LM3, MHCC-97H, and HepG2 cells transfected with *YY2* overexpression vector, as determined using western blotting. **(B)** YY2 expression level in HCC-LM3 cells transfected with *YY2* overexpression vector, as determined using immunofluorescence (scale bars: 15 μm). **(C)** mRNA expression levels of CSC markers in HepG2 cells overexpressing *YY2*, as determined by qRT-PCR. **(D and E)** Establishment of *YY2* knock-out HCC cells using CRISPR/Cas9. Sequencing results depicting the deleted region (D) and western blotting results showing YY2 protein expression level in *YY2* knock-out HCC cells (E) are shown. Cells transfected with pcCon or corresponding wild-type cells were used as controls. β-actin was used for qRT-PCR normalization and as western blotting loading control. Quantification data are shown as mean ± SD (n = 3). *P* values were calculated using two-tailed unpaired Student's *t*-test. pcCon: pcEF9-Puro. **P* < 0.05; ***P* < 0.01.

Supplementary Figure S3

A

MHCC-97H

Frequency of tumor-sphere formation	Number of cells seeded each well									Stem cell frequency	
	256	128	64	32	16	8	4	2	1	Estimate	Upper and Lower limits
pcCon	8/8	8/8	8/8	8/8	8/8	7/8	6/8	2/8	0/8	1:4	1:2−1:6
pcYY2	8/8	8/8	8/8	8/8	8/8	3/8	2/8	0/8	0/8	1:11	1:7−1:17

B

MHCC-97H

Frequency of tumor-sphere formation	Number of cells seeded each well									Stem cell frequency	
	256	128	64	32	16	8	4	2	1	Estimate	Upper and Lower limits
WT	8/8	8/8	8/8	8/8	8/8	6/8	5/8	2/8	0/8	1:4	1:3−1:9
YY2-KO	8/8	8/8	8/8	8/8	8/8	8/8	8/8	6/8	2/8	1:1	1:1− 1:2

C

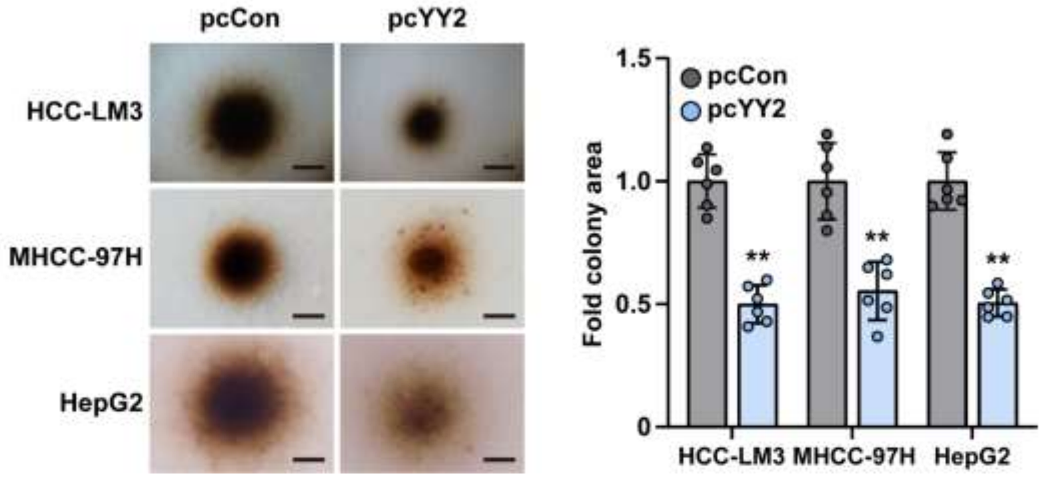

Figure S3. YY2 negatively alters liver CSC frequency. (A and B) Liver CSC frequency in *YY2*-overexpressed (A) and *YY2* knock-out (B) MHCC-97H cells, as determined by *in vitro* LDA. **(C)** Anchorage-independent colony formation potential of *YY2*-overexpressed HCC cells, as determined using soft agar assay. Representative images (scale bars: 100 μm) and quantification results (n = 6) are shown. Cells transfected with pcCon or wild-type cells were used as controls. Quantification data are shown as mean ± SD. *P* values were calculated using two-tailed unpaired Student's *t*-test. pcCon: pcEF9-Puro. $^{**}P < 0.01$.

Supplementary Figure S4

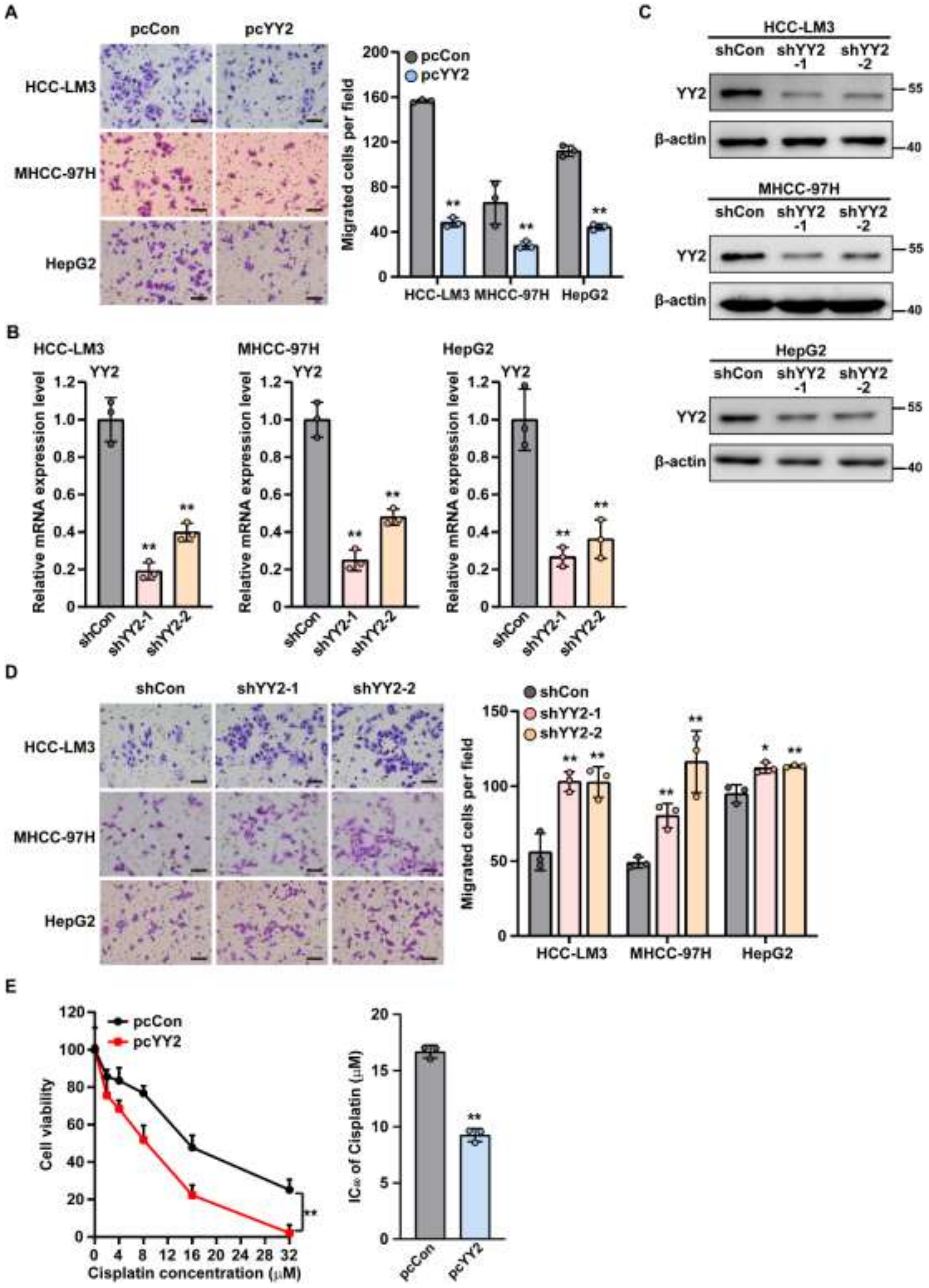

Figure S4. Effect of *YY2* alteration on HCC cells migration potential and drug

resistance. (A) Migration potential of HCC cells overexpressing *YY2*. Representative images (scale bars: 100 μm) and quantification of migrated cells from three independent experiments (n = 6/experiment) are shown. **(B and C)** YY2 mRNA (n = 3; B) and protein (C) expression levels in HCC cells transfected with shRNAs targeting different sites of YY2, as determined using qRT-PCR and western blotting, respectively. **(D)** Migration potential of *YY2* knock-down HCC cells. Representative images (scale bars: 100 μm) and quantification of migrated cells from three independent experiments (n = 6/experiment) are shown. **(E)** IC_{50} of cisplatin in *YY2*-overexpressed HCC-LM3 cells (n = 3). Cells transfected with shCon or pcCon were used as controls. β-actin was used for qRT-PCR normalization and as western blotting loading control. Quantification data are shown as mean ± SD. *P* values were calculated using two-tailed unpaired Student's *t*-test. pcCon: pcEF9-Puro. **P* < 0.05; ***P* < 0.01.

Supplementary Figure S5

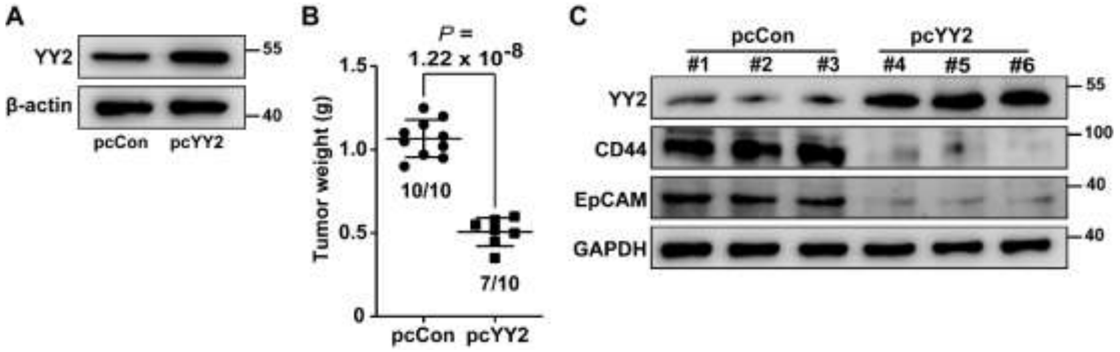

Figure S5. YY2 suppresses HCC cell stemness *in vivo*. (A) YY2 protein expression level in HCC-LM3 stably overexpressing YY2, as determined using western blotting. **(B)** Tumor weight at day 12 after transplantation. Ratio of the number of mice with tumor to the number of total mice transplanted with indicated cells are shown. **(C)** YY2, CD44, and EpCAM protein expression levels in the xenografted tumors formed using the indicated cells, as determined using western blotting. Cells transfected with pcCon were used as controls. β-actin was used as western blotting loading controls for cellular experiments; while GAPDH was used as loading controls for samples from xenograft experiments to avoid antibody cross-reactivity with mouse β-actin. Quantification data are shown as mean ± SD. *P* values were calculated using one-way ANOVA. pcCon: pcEF9-Puro.

Supplementary Figure S6

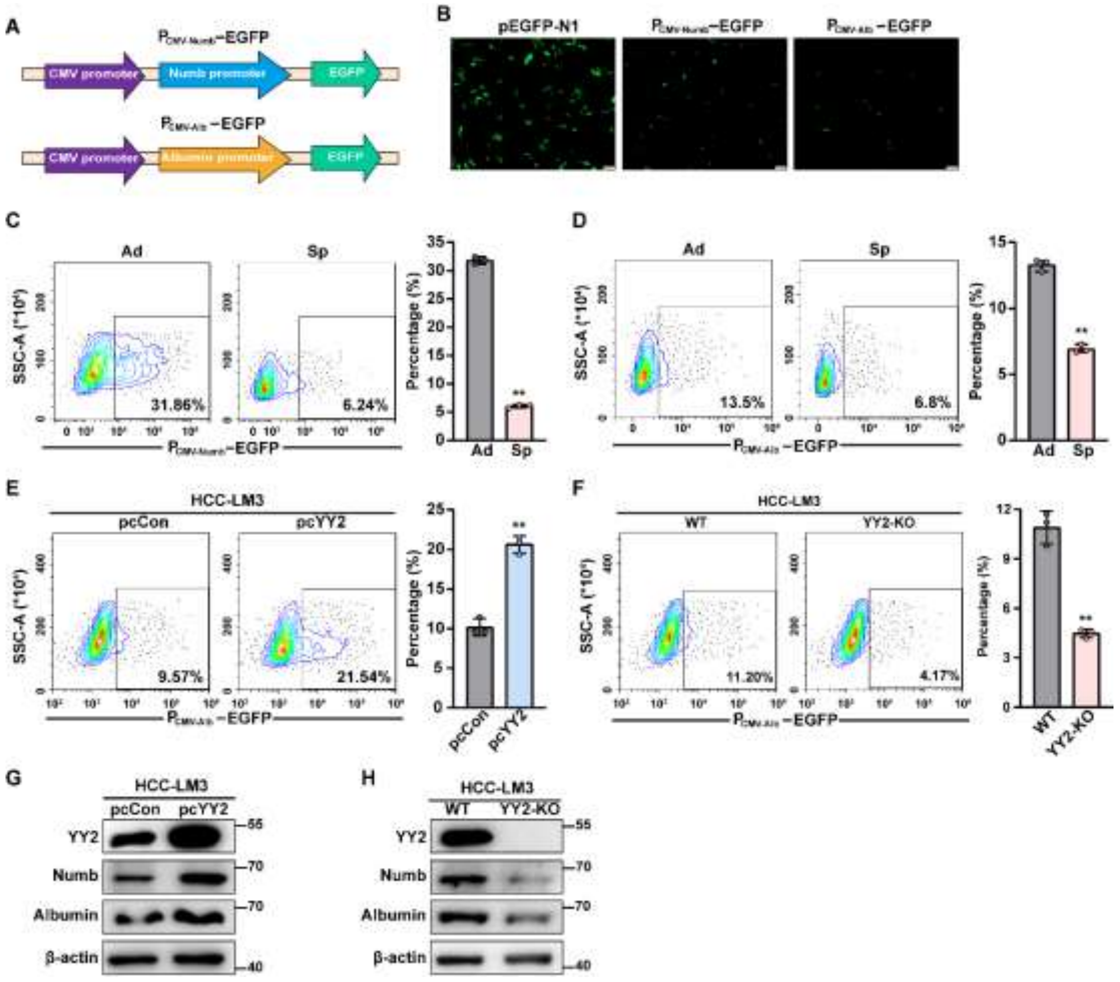

Figure S6. YY2 promotes liver CSC differentiation. (A) Schematic diagram of EGFP reporter vectors with *CMV-Numb* ($P_{CMV\text{-}Numb}$-EGFP) or *CMV-Albumin* ($P_{CMV\text{-}Alb}$-EGFP) promoter. **(B)** EGFP-positive cells in HCC-LM3 cells transfected with pEGFP-N1, $P_{CMV\text{-}Numb}$-EGFP, or $P_{CMV\text{-}Alb}$-EGFP vector (scale bars: 200 μm). **(C and D)** Percentages of EGFP-positive cells in adherent and stem-like tumor spheres formed by HCC-LM3 cells transfected with $P_{CMV\text{-}Numb}$-EGFP (C) and $P_{CMV\text{-}Alb}$-EGFP (D) vector. **(E and F)** Percentages of EGFP-positive cells in *YY2*-overexpressed (E) and *YY2* knock-out (F) HCC-LM3 cells transfected with $P_{CMV\text{-}Alb}$-EGFP vector, as analyzed using flow cytometry. **(G and H)** Protein expression levels of Numb and

Albumin in *YY2*-overexpressed (G) and *YY2* knock-out (H) HCC-LM3 cells, as determined by western blotting. Cells transfected with pcCon or wild-type cells were used as controls. β-actin was used as western blotting loading control. Ad: adherent cells; Sp: stem-like tumor spheres. Quantification data are shown as mean ± SD (n = 3). *P* values were calculated using two-tailed unpaired Student's *t*-test. pcCon: pcEF9-Puro; $^{**}P < 0.01$.

Supplementary Figure S7

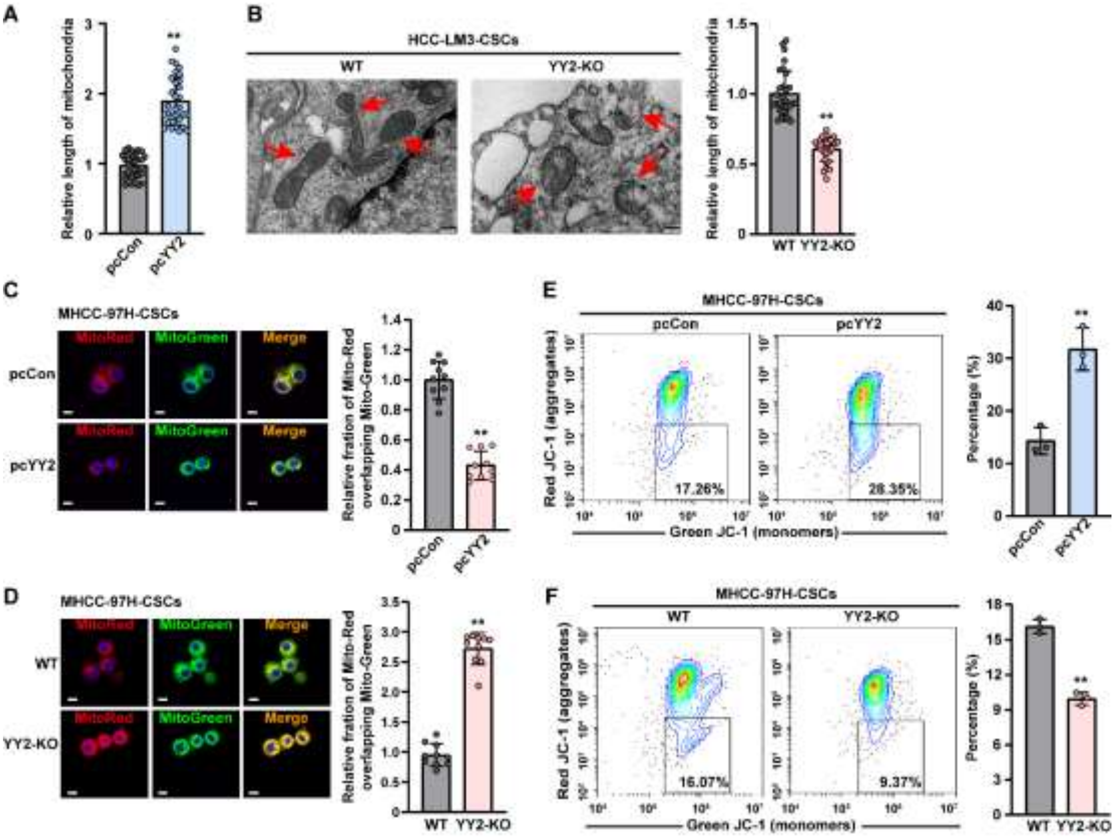

Figure S7. YY2 decreased mitochondrial membrane potential ($\Delta\Psi_m$). (A) Quantification results of mitochondria in *YY2*-overexpressed HCC-LM3 stem-like tumor spheres (n = 30). **(B)** Transmission electron microscopy images of mitochondria in *YY2* knock-out HCC-LM3 stem-like tumor spheres. Representative images (left; scale bars: 200 nm) and quantification results (right; n = 30) are shown. **(C and D)** $\Delta\Psi_m$ of *YY2*-overexpressed (C) and *YY2* knock-out (D) MHCC-97H stem-like tumor spheres, as examined using MitoTracker Red/MitoTracker Green staining and confocal microscopy. Representative images (left; scale bars: 10 μm) and quantification results (right; n = 10) are shown. **(E and F)** $\Delta\Psi_m$ in *YY2*-overexpressed (E) and *YY2* knock-out (F) MHCC-97H stem-like tumor spheres, as determined using JC-1 staining and flow cytometry (n = 3). Cells transfected with pcCon or wild-type cells were used as controls. Quantification data are shown as mean ± SD. *P* values were calculated using two-tailed unpaired Student's *t*-test. pcCon: pcEF9-Puro; ***P* <

Supplementary Figure S8

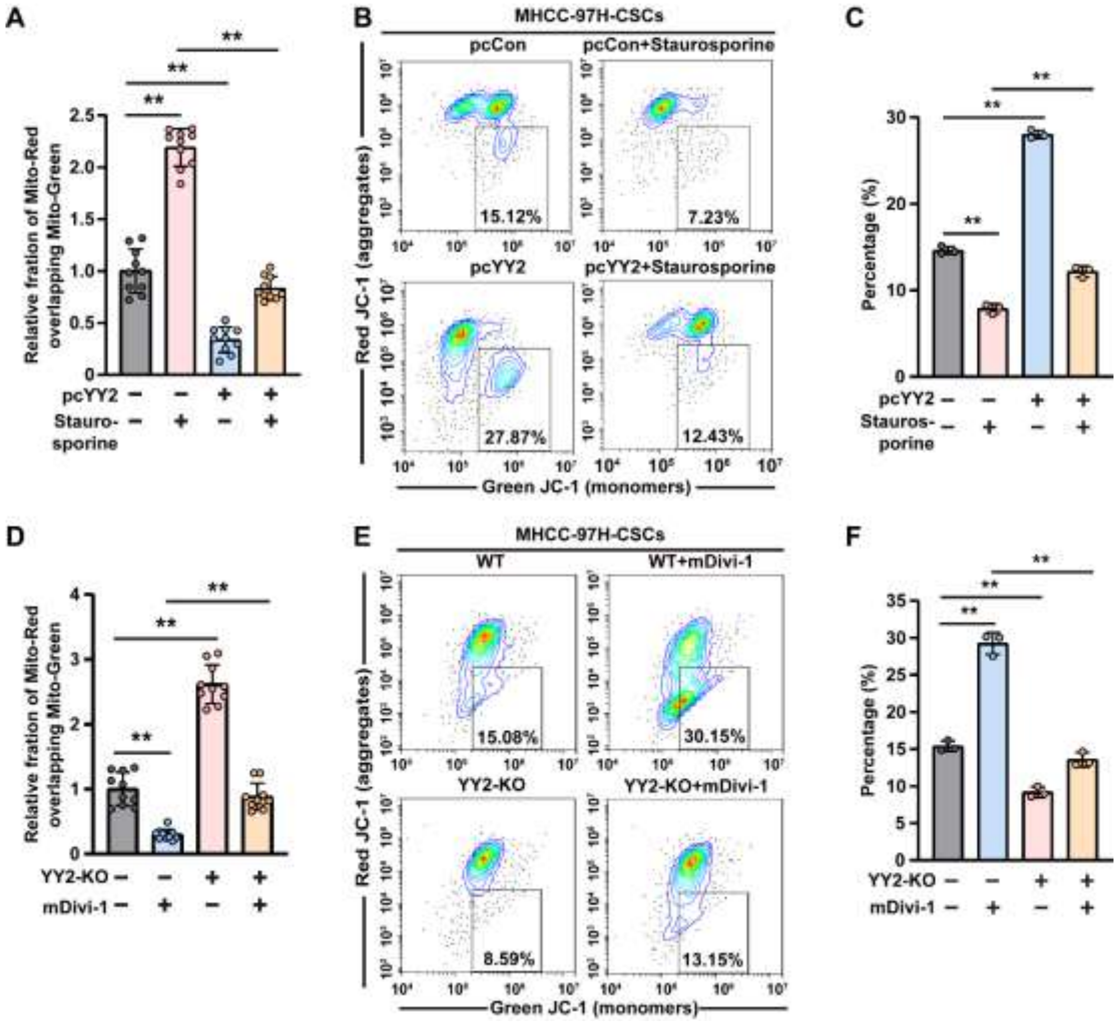

Figure S8. YY2 negatively regulates $\Delta\Psi_m$ by suppressing mitochondrial fission. (A) $\Delta\Psi$m in *YY2*-overexpressed HCC-LM3 stem-like tumor spheres treated with staurosporine, as examined using MitoTracker Red/MitoTracker Green staining (n = 10). **(B and C)** Mitochondria membrane potential in stem-like tumor spheres formed by *YY2*-overexpressed MHCC-97H cells treated with staurosporine, as determined by JC-1 staining and flow cytometry. **(D)** $\Delta\Psi$m in HCC-LM3^{YY2KO} stem-like tumor spheres treated with mDivi-1, as examined using MitoTracker Red/MitoTracker Green staining (n = 10). **(E and F)** Mitochondria membrane potential in stem-like tumor spheres formed by *YY2* knock-out MHCC-97H cells treated with mDivi-1, as determined by JC-1 staining and flow cytometry. Cells transfected with pcCon or

wild-type cells were used as controls. Final concentrations of staurosporine and mDivi-1 used were 1 μM and 10 μM, respectively. Quantification data are shown as mean ± SD. *P* values were calculated using two-tailed unpaired Student's *t*-test. pcCon: pcEF9-Puro; ***P* < 0.01.

Supplementary Figure S9

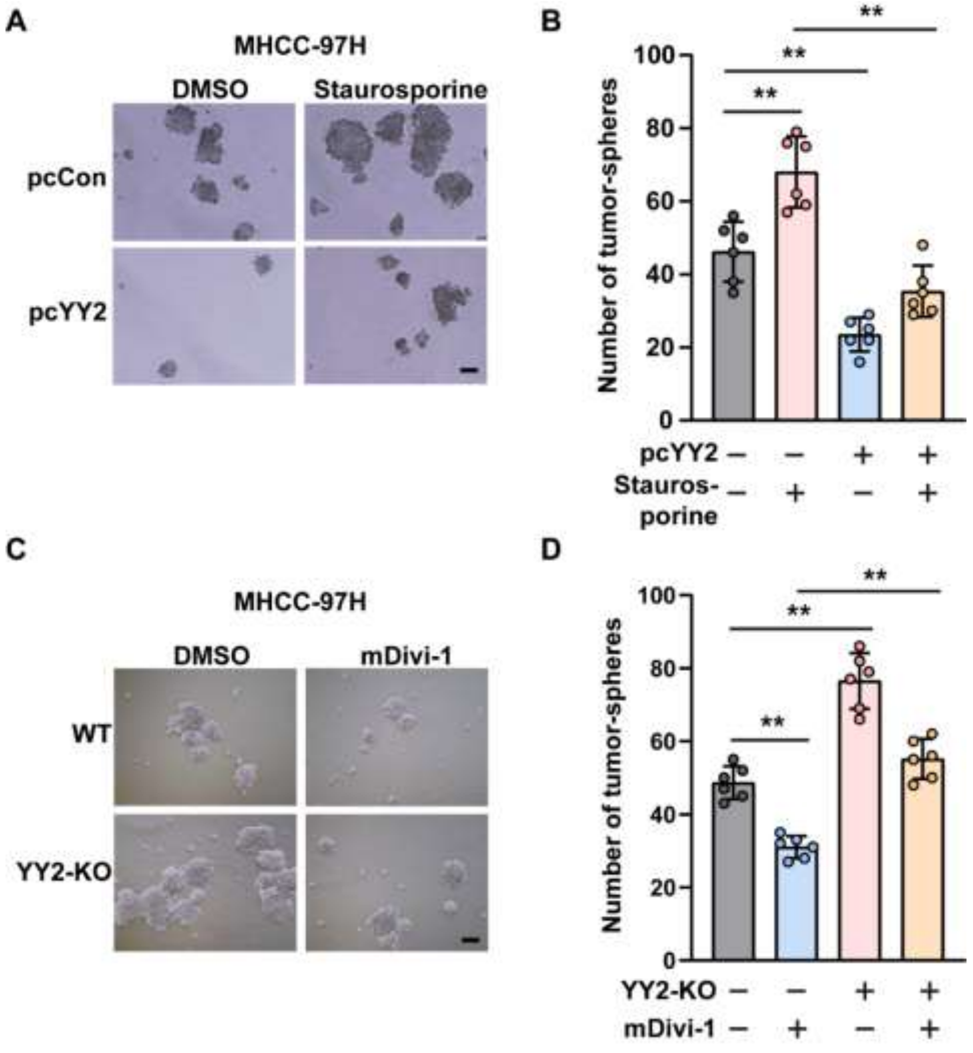

Figure S9. Mitochondrial fission is crucial for YY2 regulation on tumor sphere formation potential. (A and B) Tumor sphere formation potential of *YY2*-overexpressed MHCC-97H cells treated with staurosporine. Representative images (A) and quantification results (B) are shown. **(C and D)** Tumor sphere formation potential of MHCC-97H^{YY2KO} cells treated with mDivi-1. Representative images (C) and quantification results (D) are shown. Cells transfected with pcCon or wild-type cells were used as controls. Scale bars: 200 μm. Final concentrations of staurosporine and mDivi-1 used were 1 μM and 10 μM, respectively. Quantification data are shown as mean ± SD (n = 6). *P* values were calculated using two-tailed

unpaired Student's *t*-test. pcCon: pcEF9-Puro; $^{**}P < 0.01$.

Supplementary Figure S10

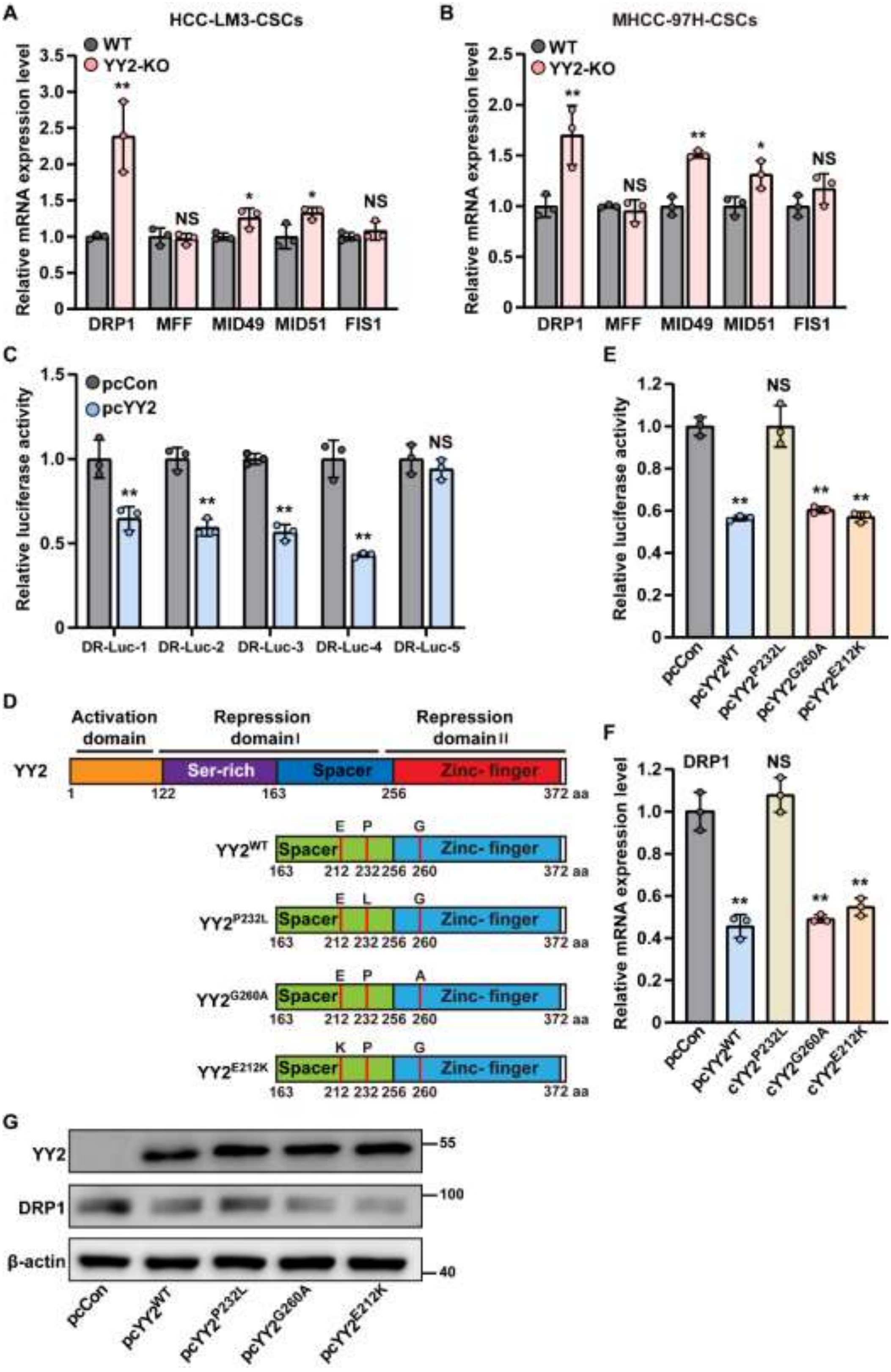

Figure S10. YY2 regulates *DRP1* transcription. (A and B) mRNA expression levels of mitochondrial fission-related genes in stem-like tumor spheres formed by *YY2*

knock-out HCC-LM3 (A) and MHCC-97H (B) cells. **(C)** Relative luciferase activities of *DRP1* reporter vectors in HCC-LM3 cells overexpressing *YY2*. **(D)** Schematic diagram showing *YY2* mutations in corresponding mutant *YY2* overexpression vectors (pcYY2^{P232L}, pcYY2^{G260A}, and pcYY2^{E212K}, respectively). **(E–G)** Relative luciferase activities of DR-Luc-1 (E), DRP1 mRNA (F), and protein (G) expression levels in HCC-LM3 cells overexpressing indicated *YY2* mutants. Cells transfected with pcCon or corresponding wild-type cells were used as controls. Quantification data are shown as mean ± SD (n = 3). *P* values were calculated using two-tailed unpaired Student's *t*-test. pcCon: pcEF9-Puro; *$P < 0.05$; **$P < 0.01$; NS: not significant.

Supplementary Figure S11

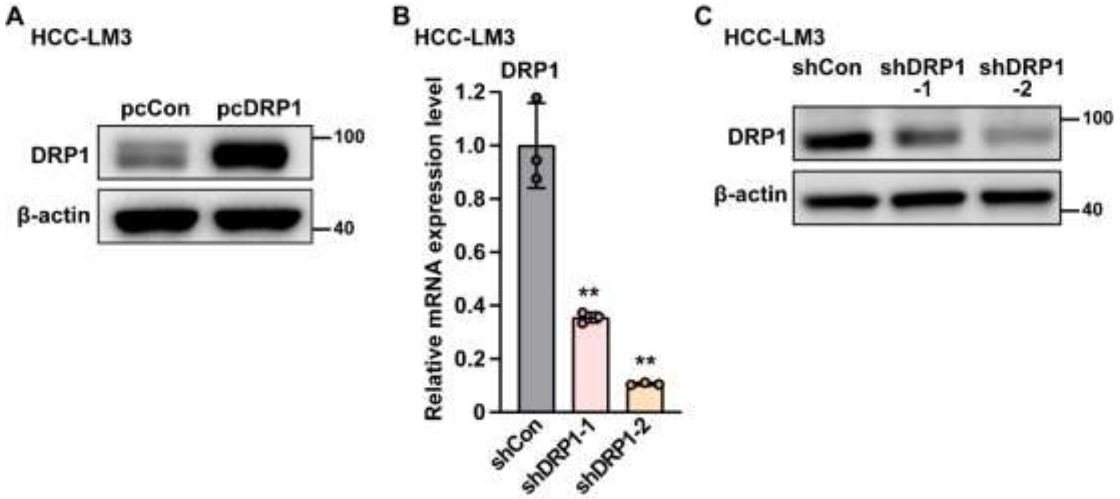

Figure S11. Efficacies of *DRP1* overexpression vector and shRNA expression vectors targeting *DRP1*. (A) DRP1 protein expression level in HCC-LM3 cells transfected with pcDRP1, as determined using western blotting. (**B and C)** DRP1 mRNA (B) and protein (C) expression levels in HCC-LM3 cells transfected with shRNAs targeting different sites of *DRP1*, as determined using qRT-PCR and western blotting, respectively. Cells transfected with pcCon or shCon were used as controls. β-actin was used for qRT-PCR normalization and as western blotting loading control. Quantification data are shown as mean ± SD (n = 3). *P* values were calculated using two-tailed unpaired Student's *t*-test. pcCon: pcEF9-Puro; ***P* < 0.01.

Supplementary Figure S12

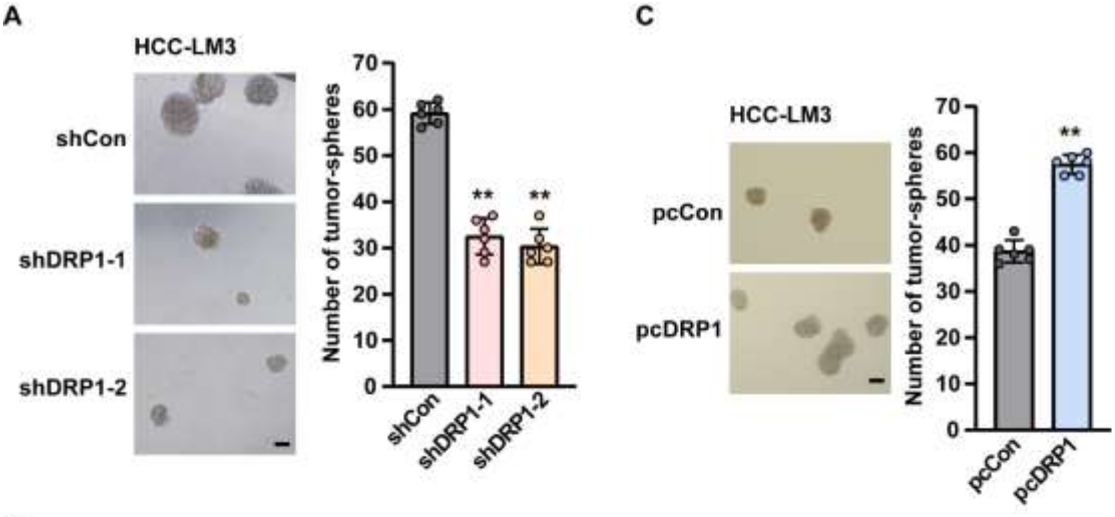

B

HCC-LM3

Frequency of tumor-sphere formation	Number of cells seeded each well									Stem cell frequency	
	256	128	64	32	16	8	4	2	1	Estimate	Upper and Lower limits
shCon	8/8	8/8	8/8	8/8	8/8	7/8	5/8	3/8	0/8	1:4	1:3−1:7
shDRP1-1	8/8	8/8	8/8	8/8	7/8	4/8	0/8	0/8	0/8	1:13	1:8−1:21
shDRP1-2	8/8	8/8	8/8	8/8	7/8	3/8	0/8	0/8	0/8	1:14	1:9−1:22

D

HCC-LM3

Frequency of tumor-sphere formation	Number of cells seeded each well									Stem cell frequency	
	256	128	64	32	16	8	4	2	1	Estimate	Upper and Lower limits
pcCon	8/8	8/8	8/8	8/8	8/8	7/8	5/8	3/8	0/8	1:4	1:3−1:7
pcDRP1	8/8	8/8	8/8	8/8	8/8	8/8	8/8	7/8	5/8	1:1	1:1−1:2

Figure S12. DRP1 promotes HCC cells stemness. (A and B) Tumor sphere formation potential (A; scale bars: 200 μm; n = 6) and liver CSC frequency (B) in *DRP1* knock-down HCC-LM3 cells, as determined using *in vitro* LDA. **(C and D)** Tumor sphere formation potential (C; scale bars: 200 μm; n = 6) and liver CSC frequency (D) in *DRP1*-overexpressed HCC-LM3 cells, as determined using *in vitro* LDA. Cells transfected with shCon or pcCon were used as controls. Quantification data are shown as mean ± SD. *P* values were calculated using two-tailed unpaired Student's *t*-test. pcCon: pcEF9-Puro; ***P* < 0.01.

Supplementary Figure S13

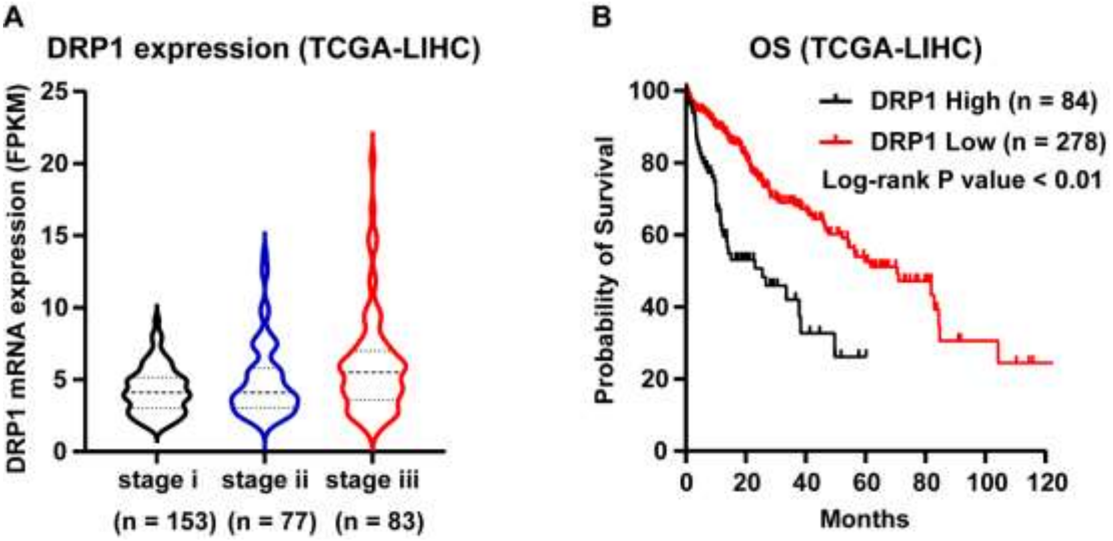

Figure S13. DRP1 expression level positively correlates with disease progression and poor prognosis in clinical HCC patients. (A) Correlation between DRP1 expression level and HCC disease progression, as analyzed using TCGA dataset (n = 313; P < 0.05). **(B)** Kaplan-Meier plot of overall survival (OS) in clinical HCC patients with low and high DRP1 expression as obtained from the TCGA database (n = 362; P < 0.01). P values were calculated using one-way ANOVA. LIHC: liver hepatocellular carcinoma.

Supplementary Figure S14

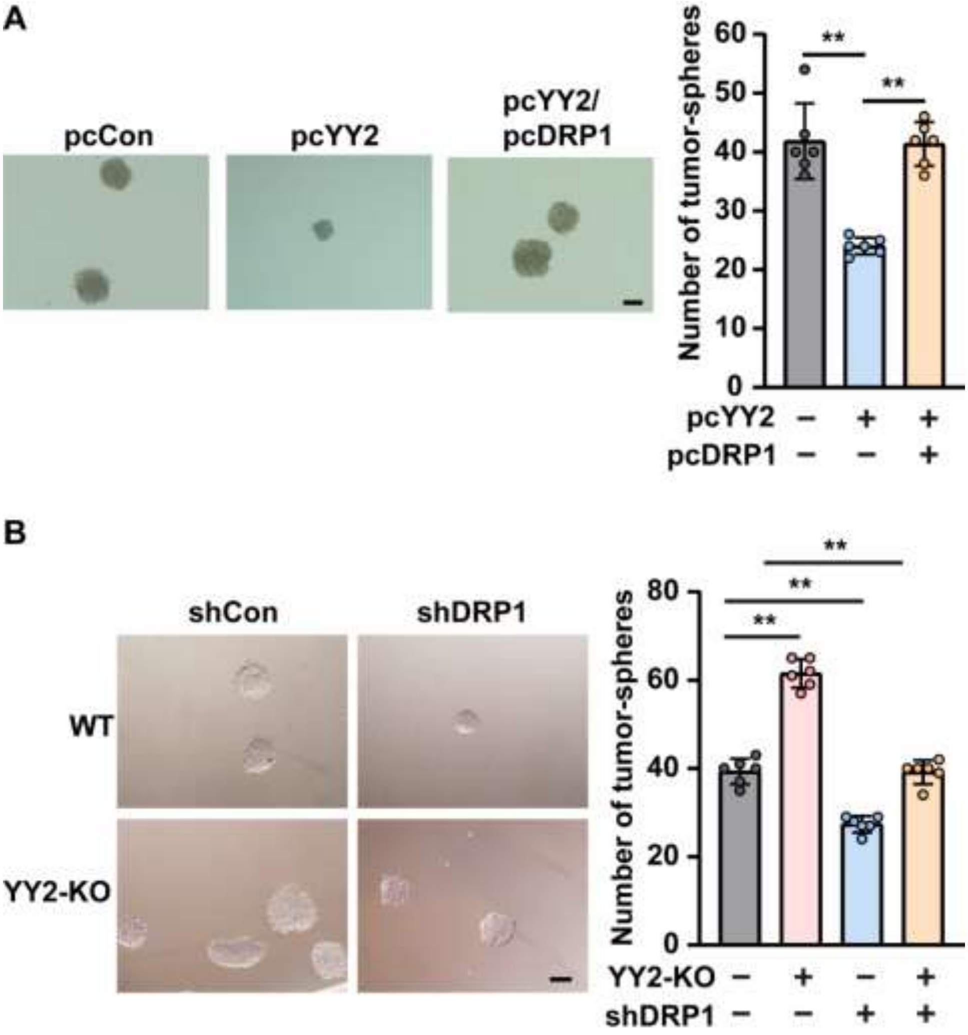

Figure S14. DRP1 downregulation is crucial for YY2 regulation on stem-like tumor sphere formation potential. (A and B) Tumor sphere formation potential of *YY2*-overexpressed, *DRP1*-overexpressed (A) and *YY2* knock-out, *DRP1* knock-down (B) HCC-LM3 cells. Cells transfected with pcCon or wild-type cells transfected with shCon were used as controls. Scale bars: 200 μm. Quantification data are shown as mean ± SD (n = 6). *P* values were calculated using two-tailed unpaired Student's *t*-test. pcCon: pcEF9-Puro. ***P* < 0.01.

Supplementary Figure S15

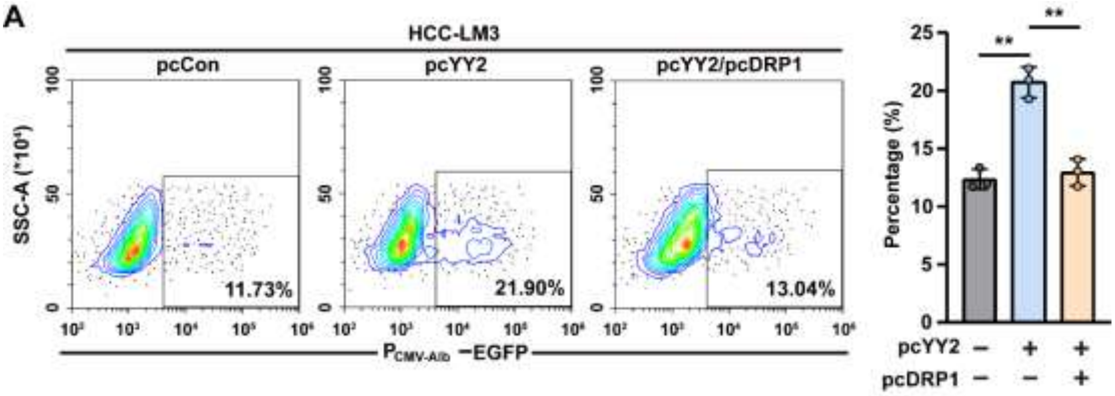

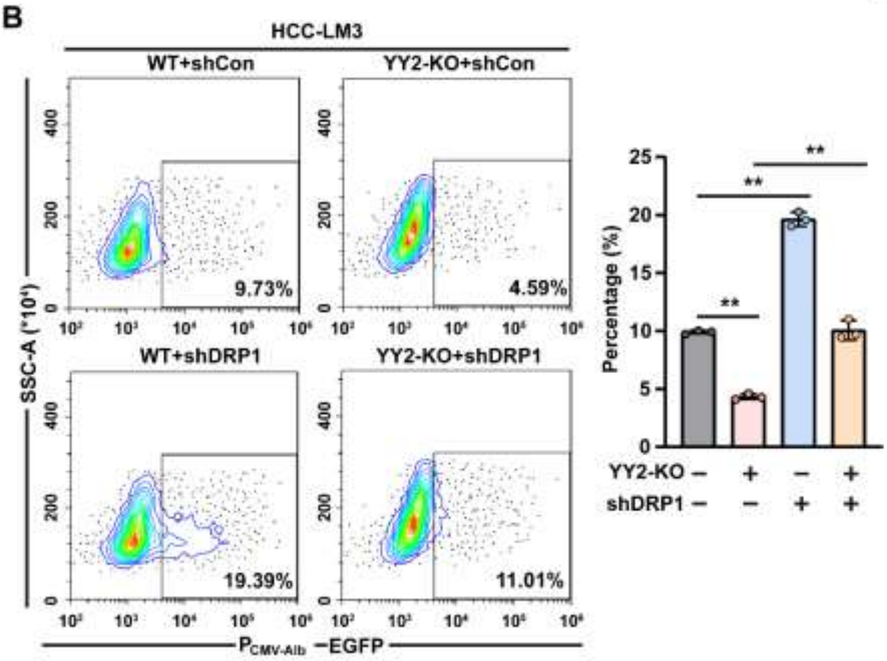

Figure S15. YY2 promotes liver CSC differentiation by suppressing DRP1. (A and B) Percentages of EGFP-positive cells in *YY2*-overexpressed, *DRP1*-overexpressed (A) and *YY2* knock-out, *DRP1* knock-down (B) HCC-LM3 cells transfected with $P_{CMV-Alb}$-EGFP vector, as analyzed using flow cytometry. Cells transfected with pcCon or wild-type cells transfected with shCon were used as controls. Quantification data are shown as mean ± SD (n = 3). *P* values were calculated using two-tailed unpaired Student's *t*-test. pcCon: pcEF9-Puro; ***P* < 0.01.

Supplementary Figure S16

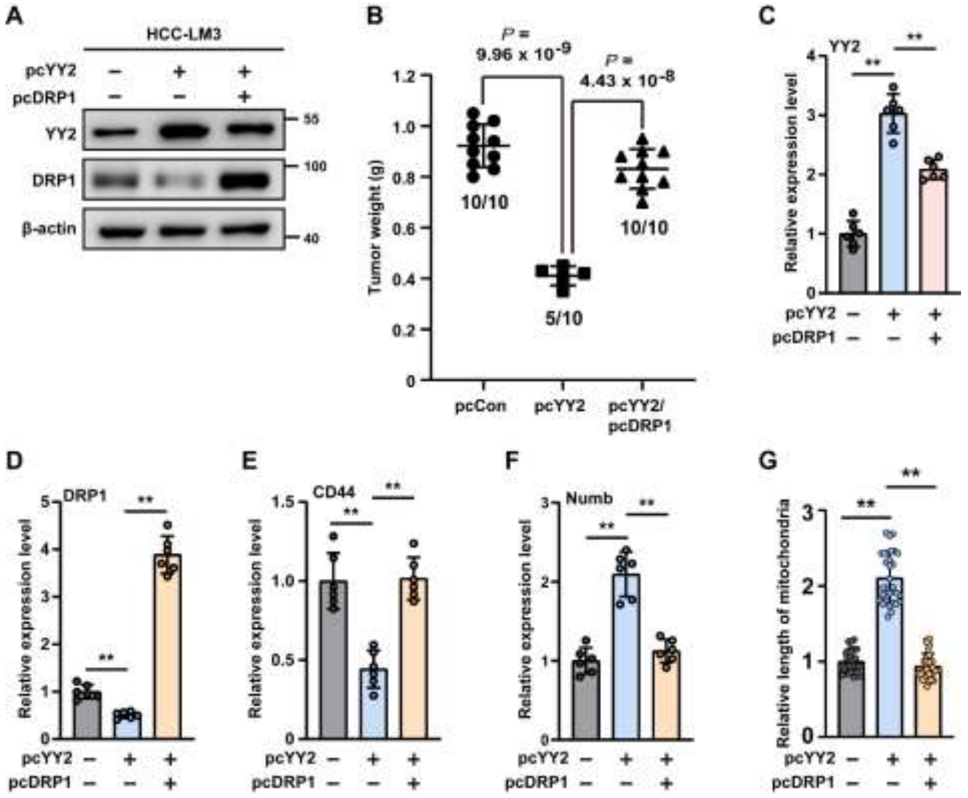

Figure S16. YY2 mediates HCC tumorigenesis potential by regulating DRP1. (A) Protein expression levels of YY2 and DRP1 in *YY2*-overexpressed, *DRP1*-overexpressed HCC-LM3 stable cells, as examined using western blotting. **(B)** Tumor weight at day 14 after transplantation. Ratio of the number of mice with tumor to the number of total mice transplanted with indicated cells are shown. **(C–F)** Expression levels of YY2 (C), DRP1 (D), CD44 (E), and Numb (F) in the tissue section of xenografted tumors formed by the indicated cells, as analyzed using immunohistochemical staining (n = 6). **(G)** Relative length of mitochondria in the tissue section of xenografted tumors formed by the indicated cells, as analyzed using transmission electron microscopy (n = 30). Tumor lesions formed by HCC-LM3 cells transfected with pcCon were used as controls. β-actin was used as western blotting loading control. Quantification data are shown as mean ± SD. *P* values were calculated using one-way ANOVA (B) or two-tailed unpaired Student's *t*-test (C–G). pcCon: pcEF9-Puro. ** $P < 0.01$.

Supplementary Figure S17

A

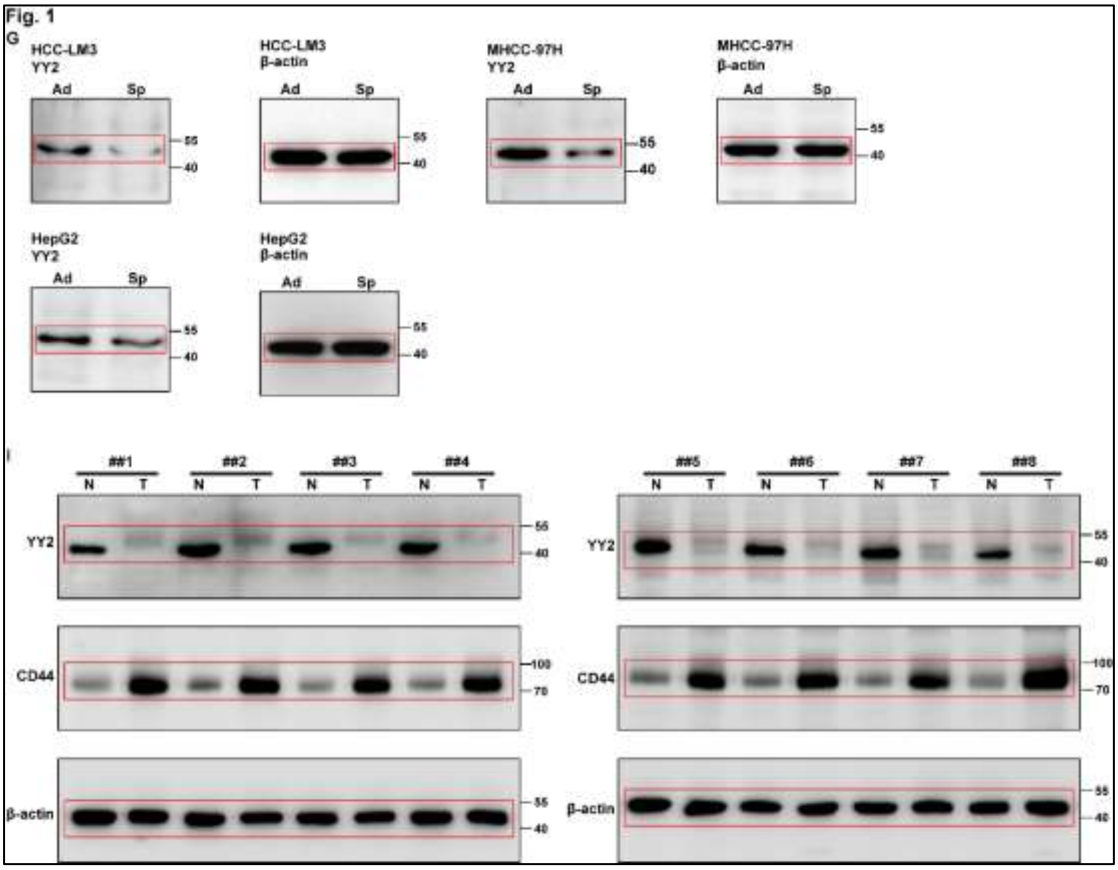

Figure S17. Uncropped western blots with the indicated areas of selection in Figs.1, 2, 5, 7 and Supplementary Figs. S2, S4, S5, S6, S10, S11, S16. (continued)

B

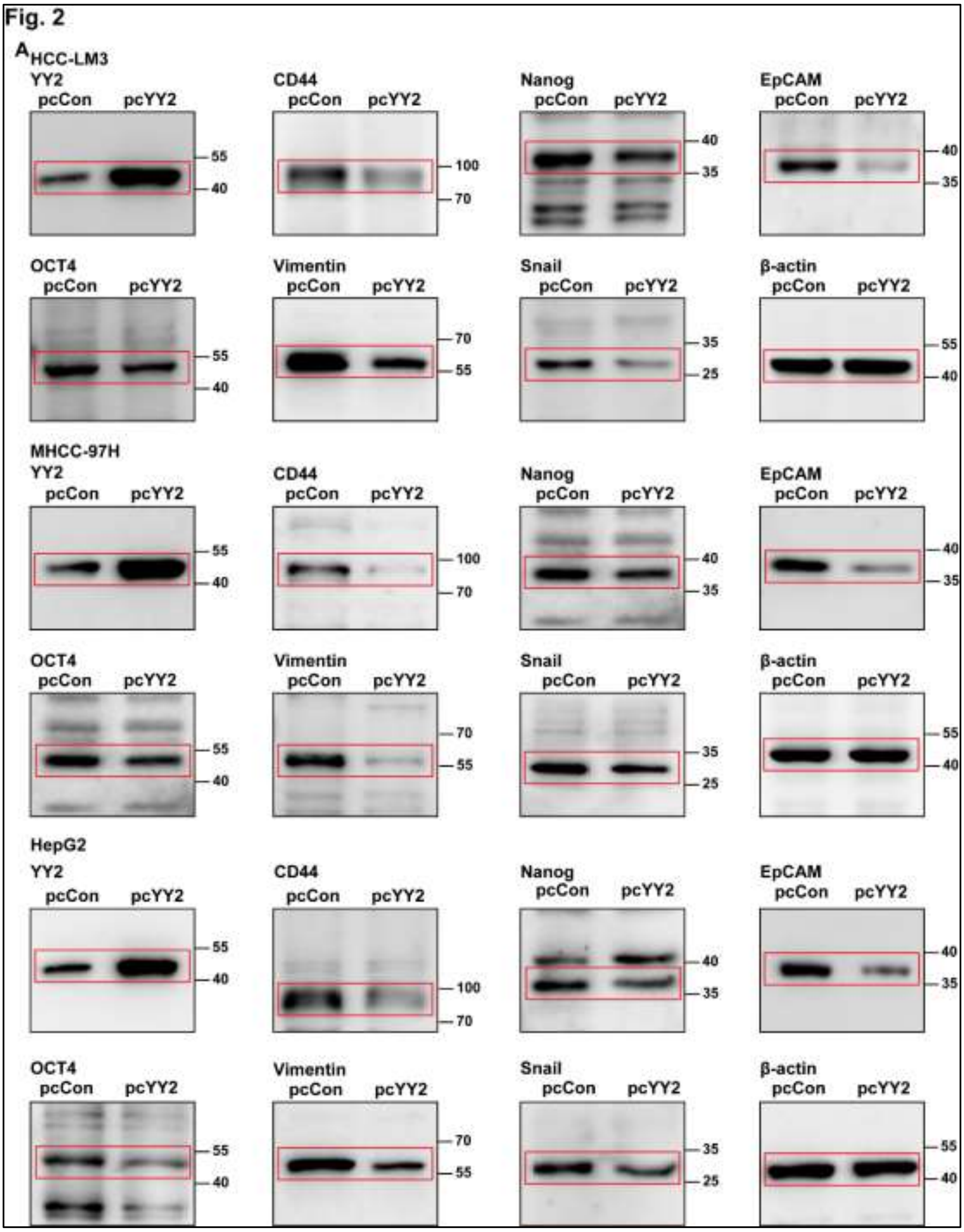

Figure S17. Uncropped western blots with the indicated areas of selection in Figs.1, 2, 5, 7 and Supplementary Figs. S2, S4, S5, S6, S10, S11, S16. (continued)

C

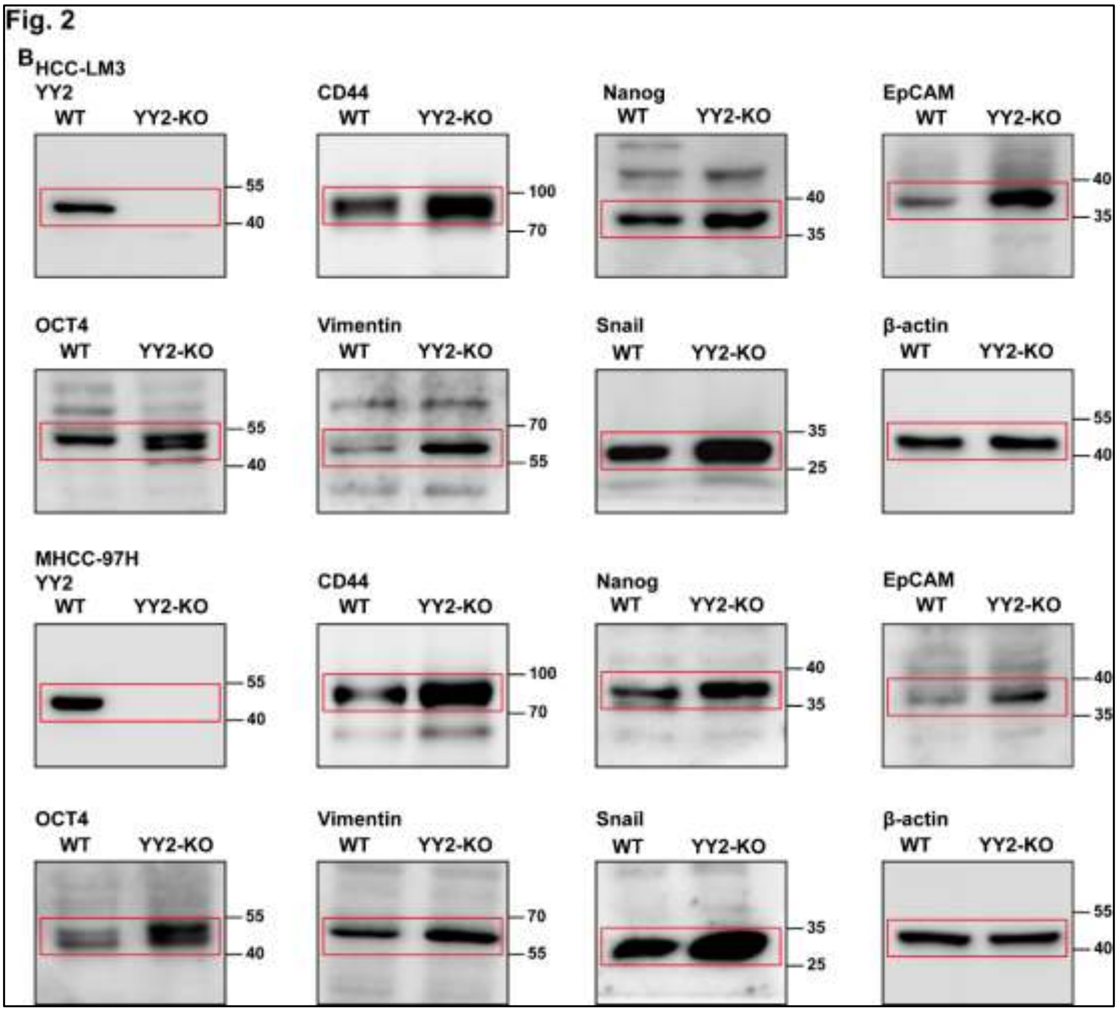

Figure S17. Uncropped western blots with the indicated areas of selection in Figs.1, 2, 5, 7 and Supplementary Figs. S2, S4, S5, S6, S10, S11, S16. (continued)

D

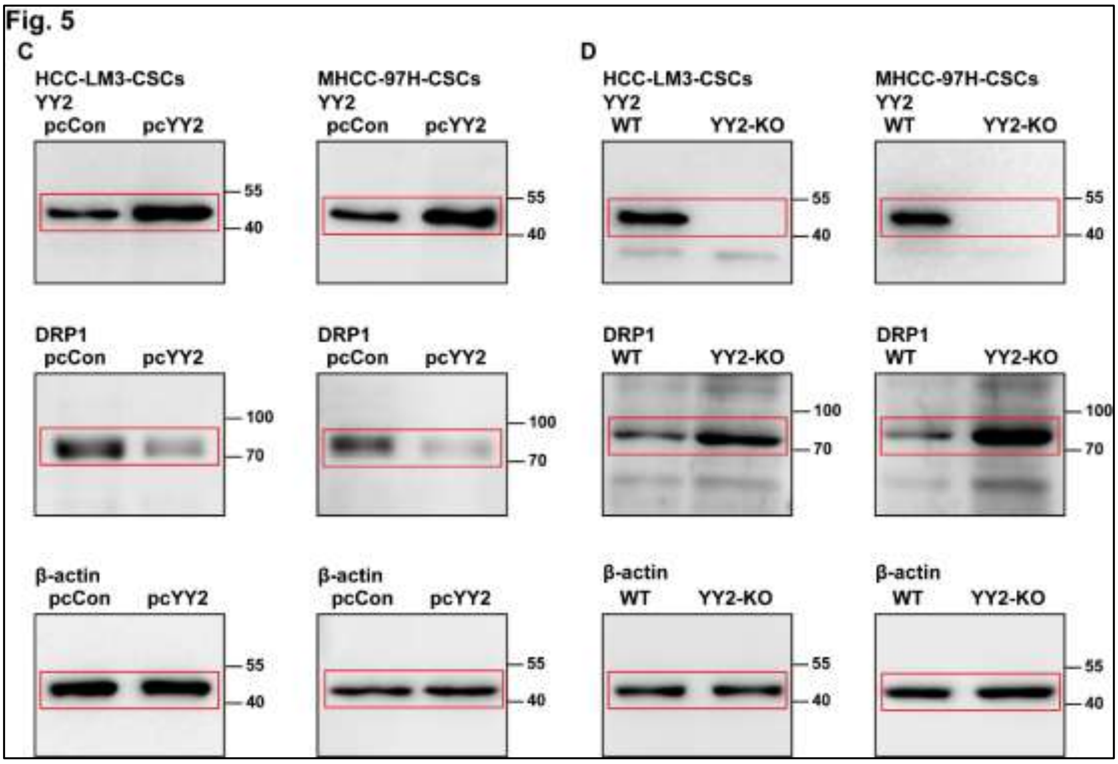

Figure S17. Uncropped western blots with the indicated areas of selection in Figs.1, 2, 5, 7 and Supplementary Figs. S2, S4, S5, S6, S10, S11, S16. (continued)

E

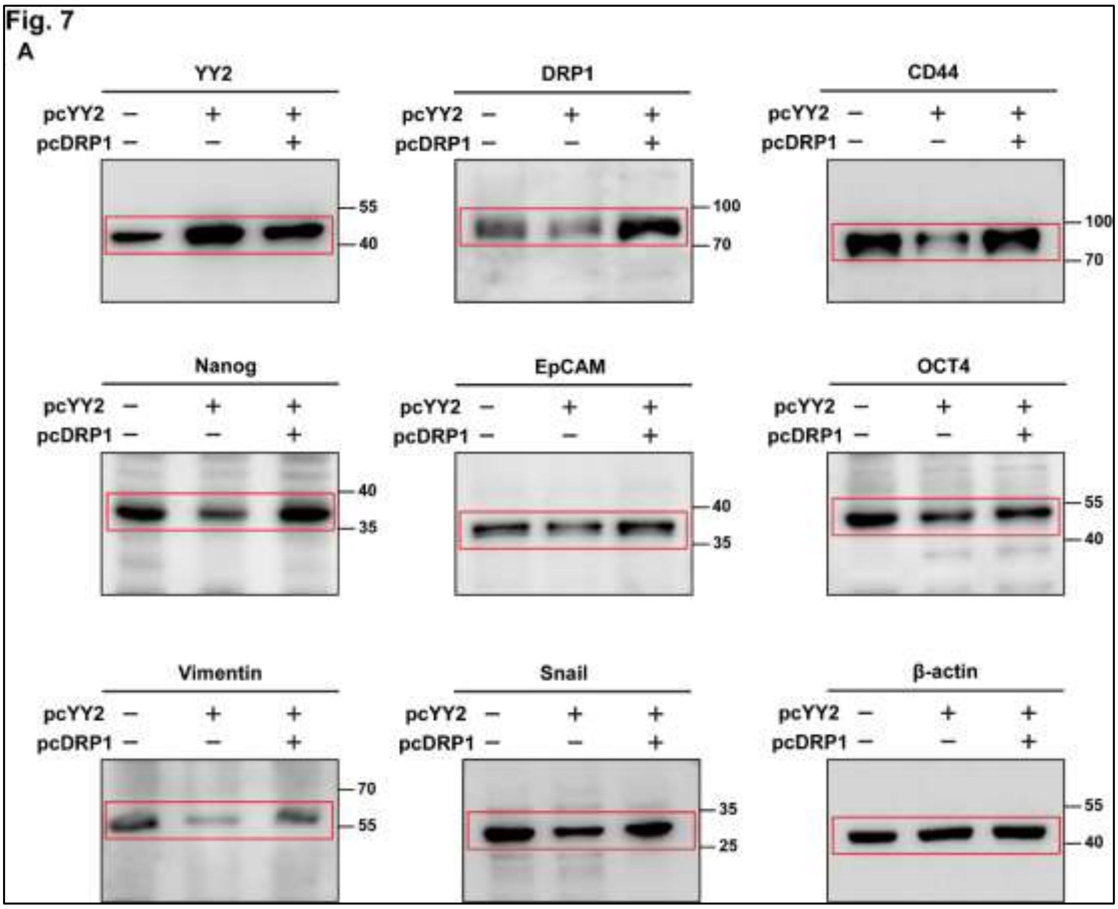

Figure S17. Uncropped western blots with the indicated areas of selection in Figs.1, 2, 5, 7 and Supplementary Figs. S2, S4, S5, S6, S10, S11, S16. (continued)

F

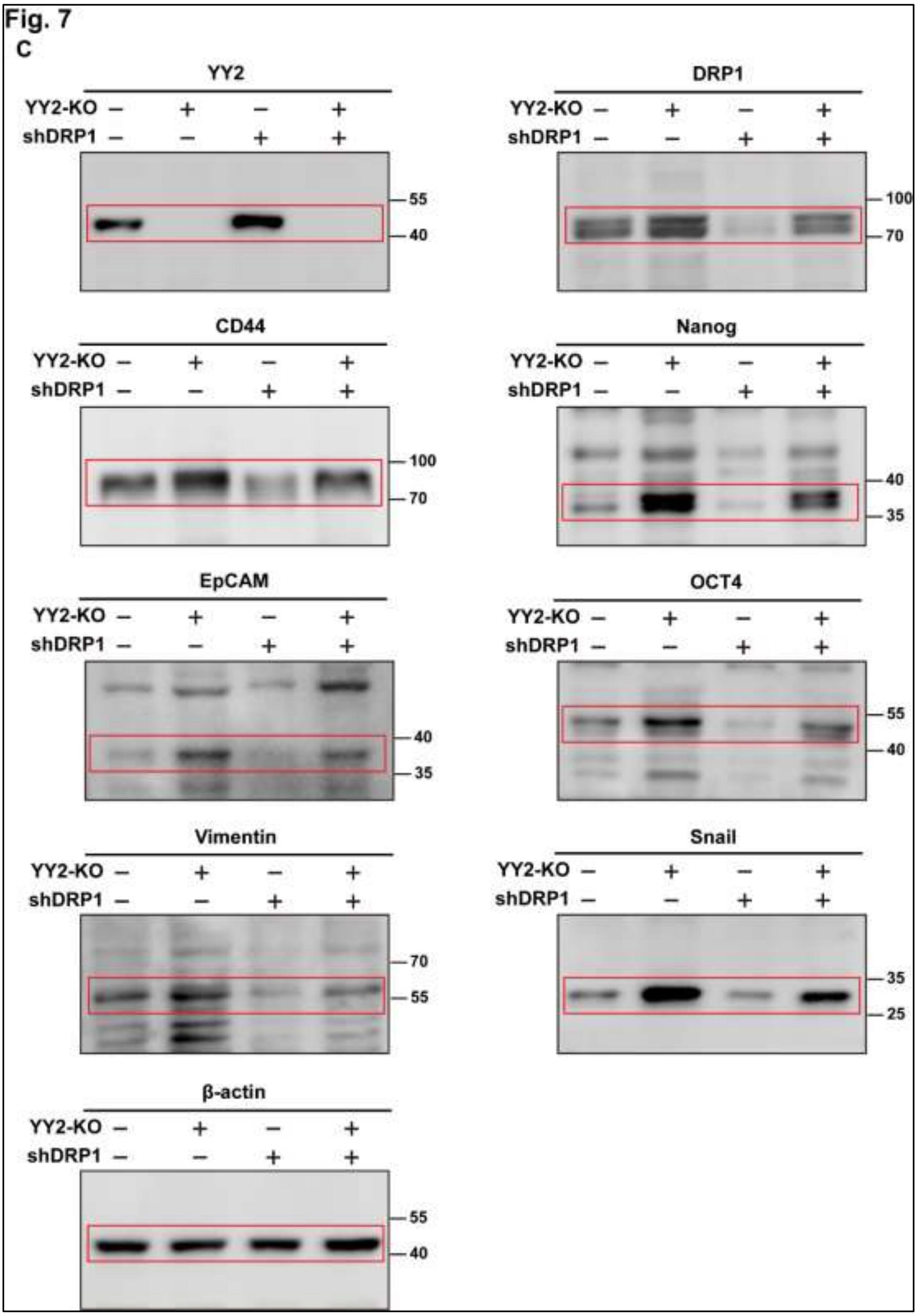

Figure S17. Uncropped western blots with the indicated areas of selection in Figs.1, 2, 5, 7 and Supplementary Figs. S2, S4, S5, S6, S10, S11, S16. (continued)

G

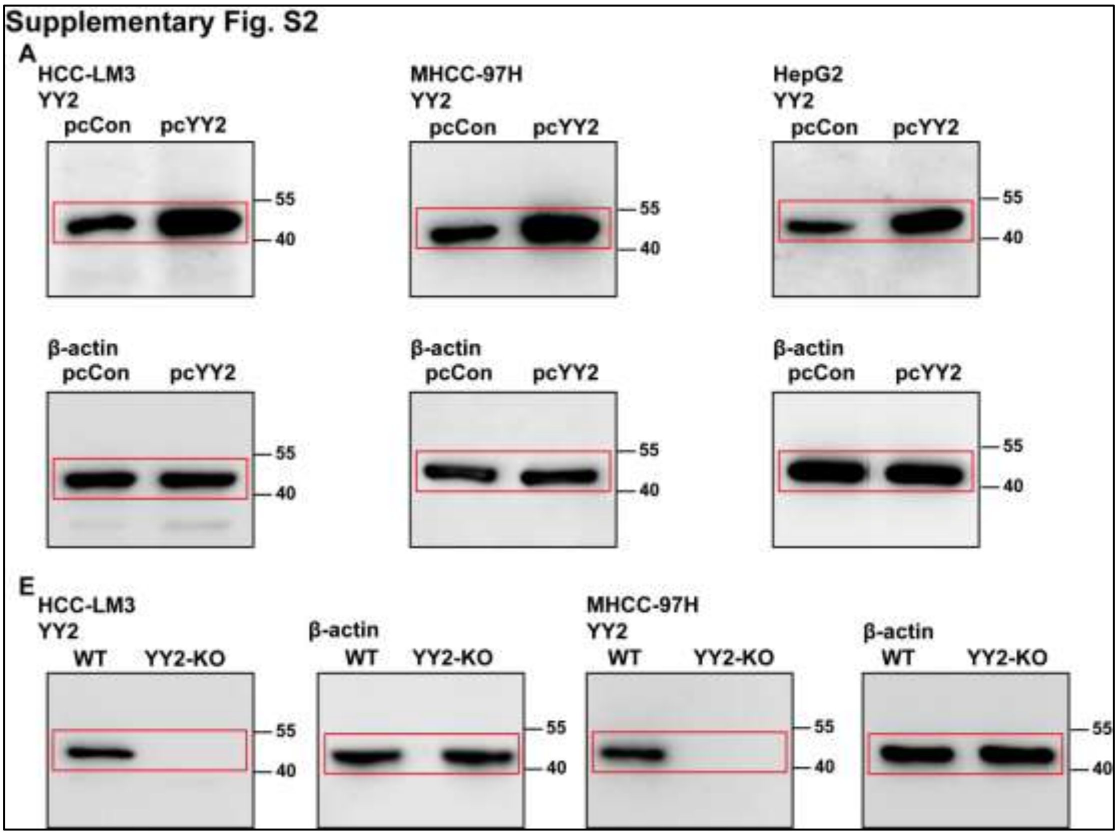

Figure S17. Uncropped western blots with the indicated areas of selection in Figs.1, 2, 5, 7 and Supplementary Figs. S2, S4, S5, S6, S10, S11, S16. (continued)

H

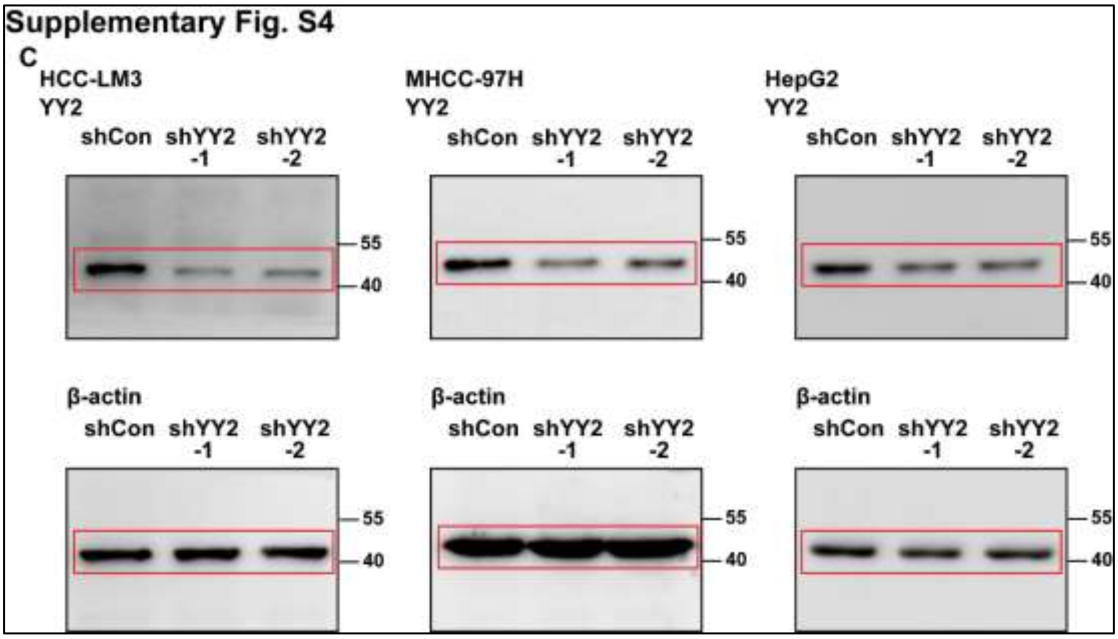

Figure S17. Uncropped western blots with the indicated areas of selection in Figs.1, 2, 5, 7 and Supplementary Figs. S2, S4, S5, S6, S10, S11, S16. (continued)

I

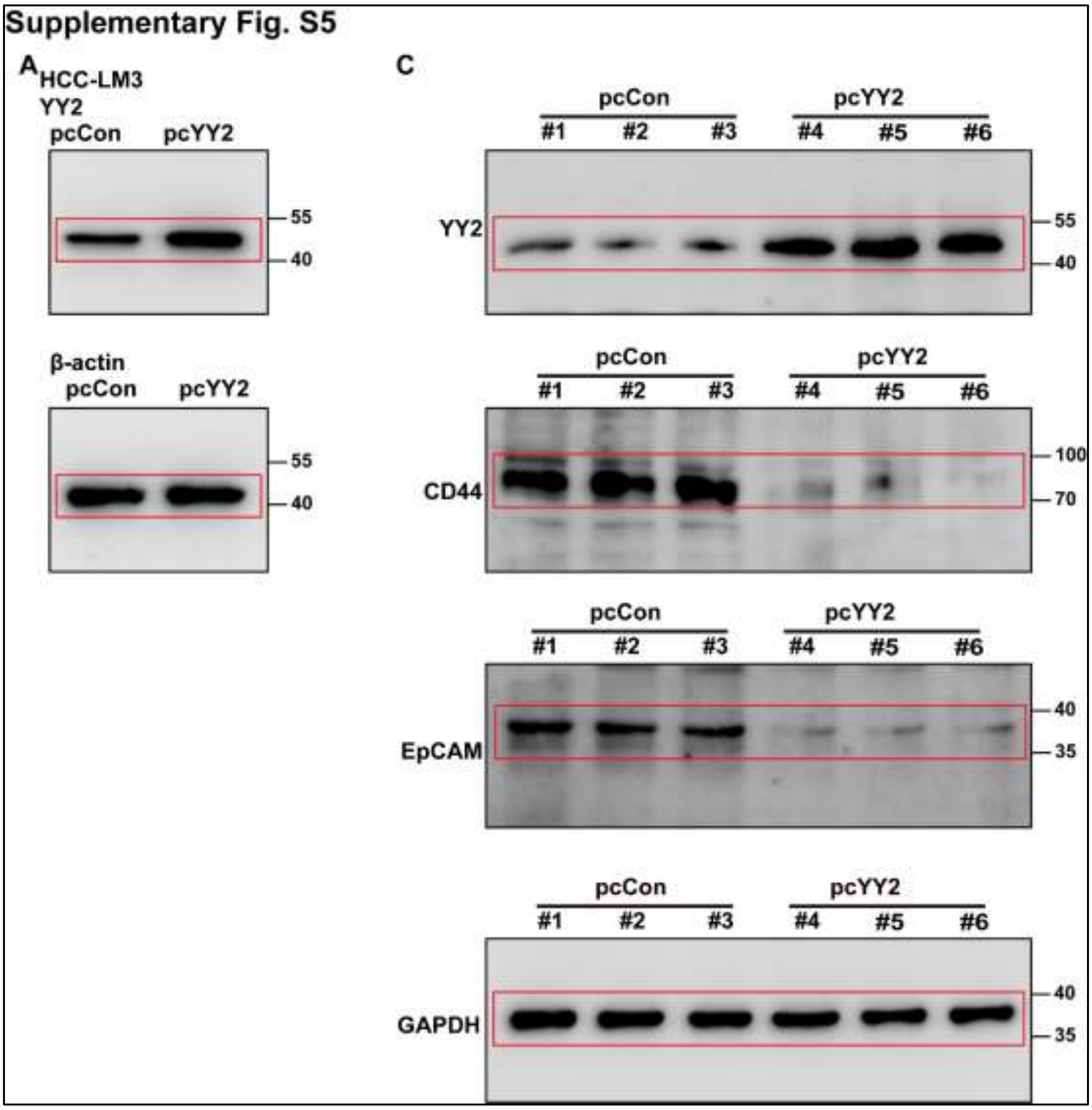

Figure S17. Uncropped western blots with the indicated areas of selection in Figs.1, 2, 5, 7 and Supplementary Figs. S2, S4, S5, S6, S10, S11, S16. (continued)

J

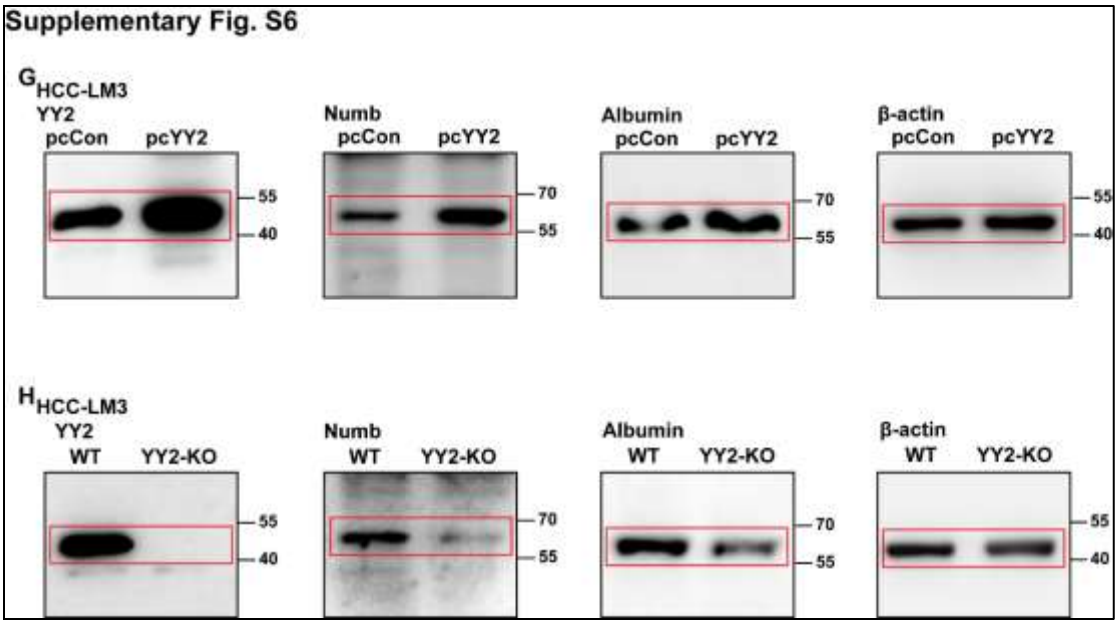

Figure S17. Uncropped western blots with the indicated areas of selection in Figs.1, 2, 5, 7 and Supplementary Figs. S2, S4, S5, S6, S10, S11, S16. (continued)

K

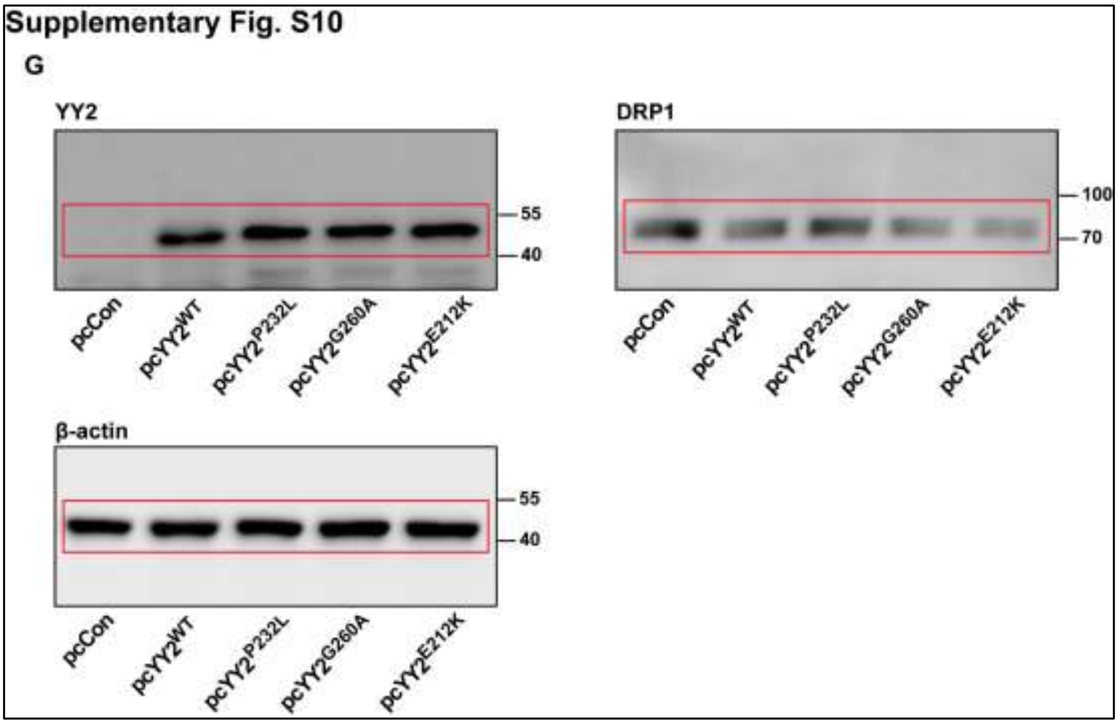

Figure S17. Uncropped western blots with the indicated areas of selection in Figs.1, 2, 5, 7 and Supplementary Figs. S2, S4, S5, S10, S11, S16. (continued)

L

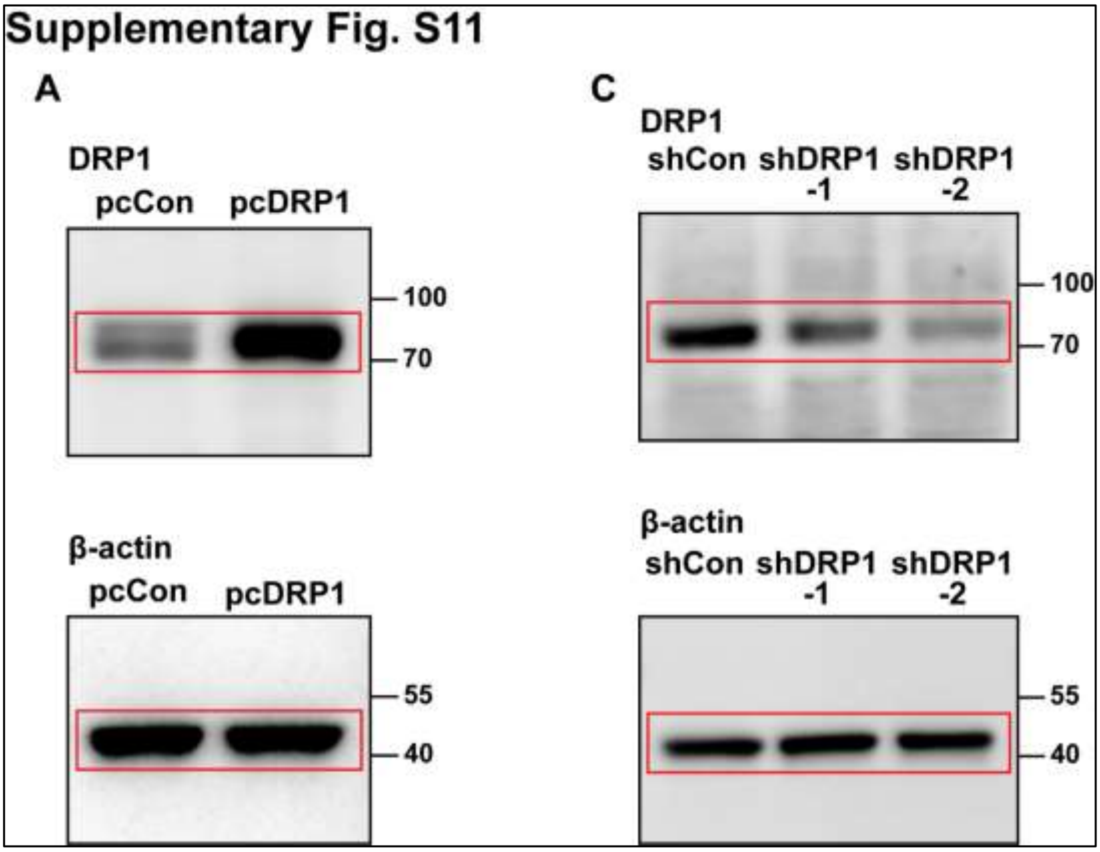

Figure S17. Uncropped western blots with the indicated areas of selection in Figs.1, 2, 5, 7 and Supplementary Figs. S2, S4, S5, S6, S10, S11, S16. (continued)

M

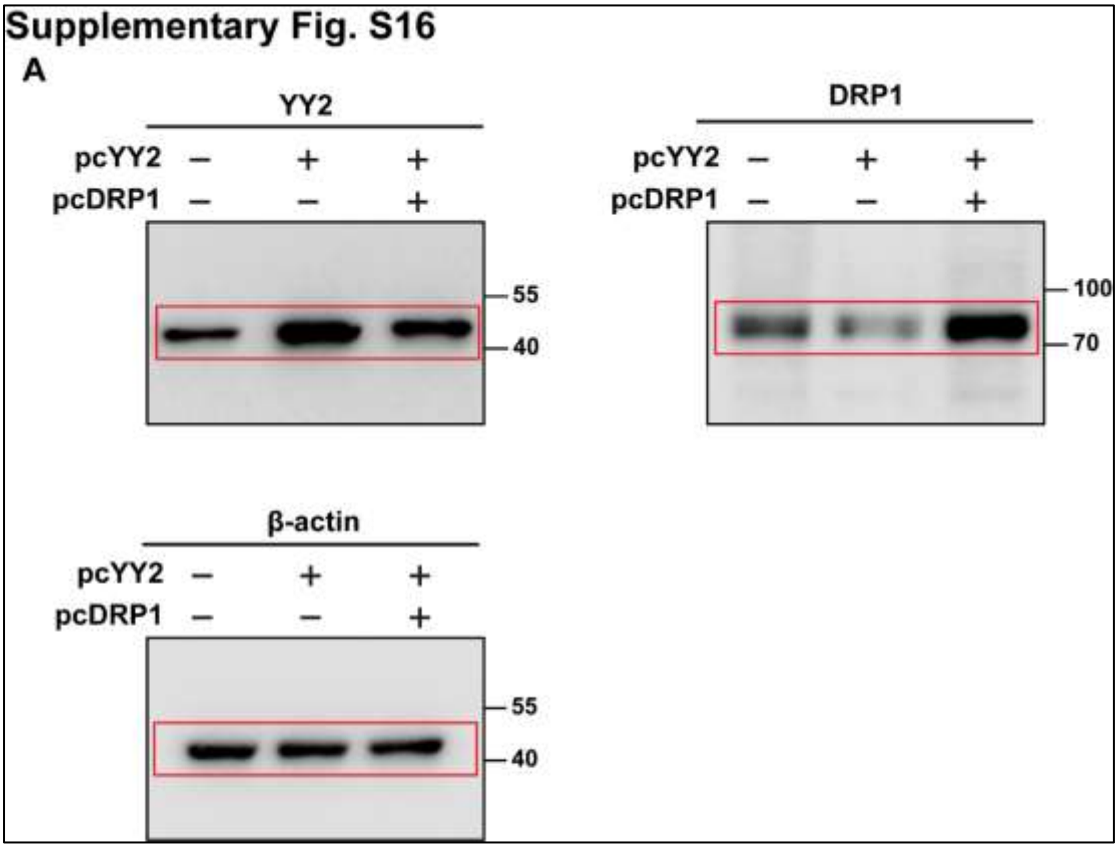

Figure S17. Uncropped western blots with the indicated areas of selection in Figs.1, 2, 5, 7 and Supplementary Figs. S2, S4, S5, S6, S10, S11, S16.

Supplementary Table S1. Primer pairs used for qRT-PCR.

**Genes**	**Refseq No.**	**Forward primer sequence (5'-3')**	**Reverse primer sequence (5'-3')**
YY2	NM_206923.4	GAAGTGGTGGGCTATTGCGA	AGGGTCATCTGGAAGTGCTC
GTF2H4	NM_001517.5	TATTGGACCGATTGTATGGGCA	AGCCCTGTACTTTCCTCCTGA
Nanog	NM_024865.4	ATAACCTTGGCTGCCGTCTC	AGCCTCCCAATCCCAAACAA
EpCAM	NM_002354.3	TGCTGGAATTGTTGTGCTGG	AAGATGTCTTCGTCCCACGC
OCT4	NM_002701.6	TGAGTAGTCCCTTCGCAAGC	TTAGCCAGGTCCGAGGATCA
Vimentin	NM_003380.5	GACGCCATCAACACCGAGTT	CTTTGTCGTTGGTTAGCTGGT
Snail	NM_005985.4	TCGGAAGCCTAACTACAGCGA	AGATGAGCATTGGCAGCGAG
DRP1	NM_012062.5	TCACCCGGAGACCTCTCATTC	GGTTCAGGGCTTACTCCCTTAT
MFF	NM_001277061.2	ACTGAAGGCATTAGTCAGCGA	TCCTGCTACAACAATCCTCTCC
MID49	NM_139162.4	ATGGCAGAGTTCTCCCAGAAA	GCCCTGTCAATGAACCGCT
MID51	NM_019008.6	CACGGCCATTGACTTTGTGC	TCGTACATCCGCTTAACTGCC
FIS1	NM_016068.3	AGCGGGATTACGTCTTCTACC	CATGCCCACGAGTCCATCTTT
β-actin	NM_001101.3	CGAGCGCGGCTACAGCTT	TCCTTAATGTCACGCACGATTT

Supplementary Table S2. Antibodies used for western blotting, immunofluorescence, immunohistochemistry, and ChIP assay.

Antibody	Product No.	Maker	Experiment	Dilution
Anti-YY2	sc-374455	Santa Cruz Biotechnology	Western blotting<br>IHC<br>Immunofluorescence<br>ChIP assay	1/1000<br>1/100<br>1/100<br>30 μg/mL cell lysate
Anti-DRP1	12957-1-AP	Proteintech	Western blotting<br>Immunofluorescence<br>IHC (clinical tissues)	1/1000<br>1/100<br>1/100
Anti-CD44	15675-1-AP	Proteintech	Western blotting<br>Immunofluorescence<br>IHC (clinical tissue)	1/1000<br>1/100<br>1/100
Anti-EpCAM	21050-1-AP	Proteintech	Western blotting<br>IHC (clinical tissue)	1/10000<br>1/500
Anti-CD44	A19020	ABclonal	Western blotting (xenograft)<br>IHC (xenograft)	1/1000<br>1/100
Anti-EpCAM	A23075	ABclonal	Western blotting (xenograft)<br>IHC (xenograft)	1/10000<br>1/500
Anti-GAPDH	AC036	ABclonal	Western blotting	1/50000
Anti-Albumin	16475-1-AP	Proteintech	Western blotting	1/5000
Anti-Numb	PA5-121867	Invitrogen	Western blotting<br>Immunofluorescence<br>IHC	0.2 μg/mL<br>5 μg/mL<br>5 μg/mL
Anti-DRP1	ab56788	Abcam	IHC (xenograft)	1/100
Anti-Nanog	14295-1-AP	Proteintech	Western blotting	1/1000
Anti-OCT4	WL03686	Wanleibio	Western blotting	1/500
Anti-Vimentin	WL01960	Wanleibio	Western blotting	1/500
Anti-Snail	WL01863	Wanleibio	Western blotting	1/1000
Anti-β-actin	60008-1- Ig	Proteintech	Western blotting	1/100000
Anti-histone H3	17168-1-AP	Proteintech	ChIP assay	30 μg/mL cell lysate
Goat anti-rabbit IgG	ZB2301	ZSGB-BIO	Western blotting	1/10000
Goat anti-mouse IgG	ZB2305	ZSGB-BIO	Western blotting	1/10000
Alexa Fluor 488 Donkey Anti-rabbit IgG	A21206	Invitrogen	Immunofluorescence	1/500
Alexa Fluor 568 Goat Anti-rabbit IgG	A11077	Invitrogen	Immunofluorescence	1/100
Alexa Fluor 568 Goat Anti-mouse IgG	A11004	Invitrogen	Immunofluorescence	1/100
